# Secure Cognitive Radio-Enabled Vehicular Communications under Spectrum-Sharing Constraints

**DOI:** 10.3390/s21217160

**Published:** 2021-10-28

**Authors:** Suneel Yadav, Anshul Pandey, Dinh-Thuan Do, Byung Moo Lee, Adão Silva

**Affiliations:** 1 Department of Electronics and Communication Engineering, Indian Institute of Information Technology Allahabad, Prayagraj 211015, India; 2 Secure Systems Research Center, Technology Innovation Institute, Abu Dhabi 9639, United Arab Emirates; anshul@ssrc.tii.ae; 3 Department of Computer Science and Information Engineering, College of Information and Electrical Engineering, Asia University, 500 Lioufeng Rd., Wufeng, Taichung 41354, Taiwan; dodinhthuan@asia.edu.tw; 4 Department of of Intelligent Mechatronics Engineering, and Convergence Engineering for Intelligent Drone, Sejong University, Seoul 05006, Korea; blee@sejong.ac.kr; 5 Instituto de Telecomunicações (IT) and Departamento de Eletrónica, Telecomunicações e Informática (DETI), University of Aveiro, 3810-193 Aveiro, Portugal; asilva@av.it.pt

**Keywords:** physical-layer security, cognitive radio vehicular networks (CRVNs), secrecy outage probability (SOP), ergodic secrecy capacity (ESC), double-Rayleigh fading channels

## Abstract

Vehicular communication has been envisioned to support a myriad of essential fifth-generation and beyond use-cases. However, the increasing proliferation of smart and intelligent vehicles has generated a lot of design and infrastructure challenges. Of particular interest are the problems of spectrum scarcity and communication security. Consequently, we considered a cognitive radio-enabled vehicular network framework for accessing additional radio spectrum and exploit physical layer security for secure communications. In particular, we investigated the secrecy performance of a cognitive radio vehicular network, where all the nodes in the network are moving vehicles and the channels between them are modeled as double-Rayleigh fading. Furthermore, adopting an underlay approach, the communication between secondary nodes can be performed by employing two interference constraint strategies at the primary receiver; (1) Strategy I: the secondary transmitter power is constrained by the interference threshold of the primary receiver, and (2) Strategy II: the secondary transmitter power is constrained by both the interference threshold of the primary receiver and the maximum transmit power of the secondary network. Under the considered strategies, we derive the exact secrecy outage probability (SOP) and ergodic secrecy capacity (ESC) expressions over double-Rayleigh fading. Moreover, by analyzing the asymptotic SOP behavior, we show that a full secrecy diversity of 1 can be achieved, when the average channel gain of the main link goes to infinity with a fixed average wiretap channel gain. From the ESC analysis, it is revealed that the ESC follows a scaling law of $\Theta \left(\ln\left(\frac{\Omega_{m}^{2}}{\Omega_{e}^{2}}\right)\right)$ for large $\Omega_{m}$ and $\Omega_{e}$, where $\Omega_{m}$ and $\Omega_{e}$ are the average channel gains of the main link and wiretap link. The numerical and simulation results verify our analytical findings.

## 1. Introduction

With the advancement in wireless communication capabilities and increasing number of sensors, an ecosystem of automated connected vehicles has evolved into a network paradigm called the Internet of Vehicles (IoV) [1,2]. Such vehicular communication networks form an integral part of 5G and beyond wireless communication technologies. Moreover, vehicular communications can help us to realize an abundance of on the move intelligent transportation system (ITS) applications, such as safer and better travel experience to the users, infotainment services, efficient traffic management, vehicle platooning etc. [3]. In addition, vehicular communications aim at realizing ubiquitous connectivity among the vehicles in a wireless manner [4]. Therefore, to support such massive connectivity with real-time network access, a substantial amount of energy and radio resources are needed. To this end, cognitive radio technology can be exploited in the vehicular communication networks to support the shared spectrum access [5,6]. The cognitive radio-enabled vehicular communications, named cognitive radio vehicular networks (CRVNs), can exploit the additional spectrum opportunities outside the IEEE 802.11p specified standard 5.9-GHz band [7]. However, such networks are susceptible to various serious security attacks as the bulk of communication occur over the open and vulnerable wireless medium [8]. The issues of mobility, cooperative infrastructure, dynamic nature of cognitive radios, and heterogeneity can further aggravate the security concerns, as these characteristics limit the implementation of the existing key-based cryptography security infrastructure [8,9]. As of late, physical-layer security (PHY-security) has arisen as an appealing way to guarantee secure wireless transmissions and to complement the existing security infrastructure further. In contrast to the key-based upper layer security mechanisms, PHY-security techniques provide secure transmissions at the physical layer by exploiting the inherent random nature of the wireless channels such as fading, interference, etc., through various coding, signal design, and signal processing approaches [10]. Therefore, this paper aims to provide a comprehensive performance analysis of PHY-security in CRVNs under spectrum-sharing constraints.

### 1.1. Related Works

PHY-security aspects have been thoroughly investigated in the literature for various network scenarios under different fading channels without considering the cognitive framework [11,12,13,14,15,16,17,18,19,20]. Furthermore, the authors in [21] have proposed a machine-learning-based method to locate the vehicles generating jamming signals by monitoring the physical channel parameters of the vehicles in the vehicular networks. Moreover, PHY-security performance in the cognitive radio networks has been explored broadly in the literature [22,23,24,25,26,27,28,29,30,31] with or without relaying scenarios. In addition, to guarantee the quality of service (QoS) at the primary receiver, these works have employed either the single-power constraint of the maximum interference tolerable limit for the primary network or the combined power constraint of the maximum interference tolerable limit for the primary network and the maximum allowable transmission power at the secondary network. For the relay-assisted cognitive radio networks, the secrecy performance of cooperative cognitive relay networks has been analyzed in [22,23,24,25,26,27] and the references therein. Specifically, the authors in [22] investigated the secrecy performance of the cooperative cognitive relay networks in the presence of direct links. The authors in [23,24] have proposed some relay selection strategies to enhance the secrecy performance of the secondary network in the cognitive relaying systems. Moreover, the authors in [25] employed external jamming techniques for improving the security of an underlay cognitive relaying systems. The authors in [26] studied the problem of residual energy maximization for the multiple eavesdropper scenario in cognitive relaying networks. Furthermore, the authors in [27] analyzed the PHY-security performance of multiple-input–multiple-output cognitive relaying networks under the impact of outdated channel estimates.

Of particular interest are the secure underlay cognitive radio networks, where the secondary transmitter communicates with the secondary receiver under the interference constraint imposed on primary receiver in the presence of active/passive eavesdropper. Specifically, the authors in [28,29,30,31,32] evaluated the secrecy performance for cognitive radio networks. For instance, the authors in [28] investigated the secrecy performance of an underlay cognitive wiretap secondary system with multiple secondary receivers and eavesdroppers by considering the joint power constraint under Rayleigh fading channels. In [29], the authors investigated the secrecy performance of multiinput, single-output, and single-eavesdropper cognitive radio networks over correlated fading channels. The authors in [30] analyzed the secrecy performance of a cognitive wiretap system with multiantenna secondary terminals under Rayleigh fading channels. Further, the authors in [31] evaluated the secrecy performance of an underlay cognitive radio system in the presence of an active eavesdropper. Furthermore, for a single-input–multiple-output system, the authors in [32] investigated the impact of outdated channel estimates on PHY-security performance for cognitive radio networks.

However, all the aforesaid studies in [28,29,30,31,32] were limited to the scenarios where the nodes in the network are stationary (i.e., fixed infrastructures); therefore, the channel between the nodes is modeled as Rayleigh fading or Nakagami-*m* fading. In fact, the nodes in the wireless communication networks can be moving while exchanging the information, e.g., mobile of people driving on road, yielding the channel between the moving nodes as cascaded Rayleigh (double-Rayleigh) fading [33,34,35]. ( It is to be emphasized that the vehicle-to-vehicle (V2V) links undergo multiple scattering phenomena and are moving in a relatively dense scattering environment; thus, from the theoretical and empirical studies, cascaded Rayleigh channel modeling is shown to be more appropriate in resembling the dynamic V2V communication links [33,34,35]). Therefore, such V2V communications-enabled cognitive radio networks are one of the most fascinating use-cases in the upcoming 5G networks, and it is very interesting to comprehensively investigate the PHY-security in CRVNs under double-Rayleigh fading channels. In this context, the secrecy performance over cascaded fading channels has been widely studied in [35,36,37,38,39,40]. Recently, the authors in [41] studied the secrecy capacity performance for vehicular communication networks. However, these works [35,36,37,38,39,40,41] were limited to the noncognitive networking setup. Moreover, the authors in [42,43] evaluated the performance of multihop cognitive radio networks over double-Rayleigh fading channels but without taking PHY-security aspects into account. Further, the authors in [44] investigated secrecy performance of CRVNs over N*Nakagami-*m* fading channels.

### 1.2. Motivation

From the aforementioned discussion, we can infer that the bulk of the works reported towards the investigation of PHY-security aspects in cognitive communication networks were limited to the scenario where the nodes are stationary. With the emerging varied form of ITS applications and user needs on the move, CRVNs have attracted great research interest. The authors in [35,36,37,38,39,40] evaluated PHY-security performance of cooperative vehicular relaying networks but without taking the spectrum-sharing cognitive framework into consideration. Moreover, the authors in [42,43] considered double-Rayleigh fading channels and evaluated the performance of cognitive radio-enabled V2V networks, but they did not emphasize the PHY-security aspects of the considered system. Therefore, exploitation of PHY-security benefits in underlay CRVNs over cascaded fading channels is still an open issue. To this end, a little effort has been directed to analyze the secrecy performance of CRVNs over N*Nakagami-*m* fading channels in [44]. Very recently, the authors in [45] analyzed the secrecy performance of cognitive radio networks over cascaded Rayleigh fading. However, there are several differences between this work and [44,45].

- In [44], the authors considered the following assumptions while analyzing the secrecy performance of CRVNs; (i) single-power constraint of the interference on the primary receiver, and (ii) N*Nakagami-*m* fading. In addition, the system’s performance was evaluated in terms of secrecy outage probability (SOP).
- In [45], the authors considered the following assumptions while investigating the PHY-security performance of CRVNs; (i) single-power constraint of the interference on the primary receiver, (ii) cascaded Rayleigh fading for the main channel (between secondary source and secondary receiver), and Rayleigh fading for both the wiretap channel (between secondary source and secondary eavesdropper) and interference channel (between secondary source and primary receiver). However, for evaluating the system’s performance, the cascaded Rayleigh fading was transformed into a Nakagami-*m* fading approximation, and assumed statistical independence among the channel gains. In addition, the performance was evaluated in terms of SOP, intercept probability, and probability of non-zero secrecy capacity.
- Different from [44,45], in this paper, we adopt the following: (i) two power control strategies at the secondary transmitter, i.e., Strategy I: single-power constraint of the interference on the primary receiver, and Strategy II: combined power constraint of the interference on the primary receiver and the maximum transmission power at the secondary transmitter, (ii) double-Rayleigh fading for all the links, and (iii) statistical dependency among the channel gains. In addition, we evaluate the secrecy performance in terms of exact SOP, asymptotic SOP, and ergodic secrecy capacity (ESC), under Strategies I and II.

In addition, the proposed work and the works presented in [46,47] explored the cognitive radio networks under vehicular communications. However, there are several key differences between this work and [46,47], whose brief detail is as follows.

- This paper and the work presented in [46] consider all the nodes are equipped with single antenna, whereas the authors in [47] considered the secondary transmitter and primary receiver are equipped with single antenna and secondary receiver and eavesdropper are enabled with multiple antennas.
- In this work, we consider the channel between the moving vehicles to be quasistationary for a short duration (i.e., one fading block time), where the distance between the nodes is much greater than the scattering radii. Consequently, assuming the radio propagation between two moving vehicles undergoes independent double scattering events, the channel can be modeled as double-Rayleigh fading. By contrast, the works presented in [46,47] considered the scenario where the symbol period of the detected signal is larger than the coherence time of the channel, and hence, the system fading links can be characterized as time-selective. Particularly, the works [46,47] considered Rayleigh fading channels and Nakagami-*m* fading channels, respectively.
- In [46], the authors considered a single-power constraint of the interference on the primary receiver, whereas in [47], the authors adopted a combined power constraint of the interference on the primary receiver and the maximum transmission power at the secondary transmitter. Different from [46,47], this paper adopts both single-power- and combined-power-based control strategies for managing interference at the primary receiver.
- In [46], the authors evaluated the SOP and intercept probability expressions over Rayleigh fading channels, while in [47], the authors derived the expressions for the SOP and ESC over Nakagami-*m* fading channels. In this paper, we derived the SOP and ESC expressions over double-Rayleigh fading channels.

Therefore, the above differences make the contributions and results of this work fundamentally very different from [44,45,46,47]. Hence, this motivates us to develop a thorough and comprehensive investigation on the secrecy performance of CRVNs under both single and combined interference power constraints in the presence of double-Rayleigh fading channels.

### 1.3. Contributions

From the aforesaid discussion, it is obvious that there is a lack of PHY-security performance evaluation in CRVNs over double-Rayleigh fading channels by employing two power control strategies at the secondary transmitter vehicle, i.e., (1) Strategy I: where the transmit power of the secondary transmitter vehicle is only constrained by the interference threshold of the primary receiver vehicle, and (2) Strategy II: where the transmit power of the secondary transmitter vehicle is constrained by both the maximum transmit power and the interference threshold of the primary receiver vehicle. The analytical outcomes reported in this paper thus (1) face several mathematical challenges and complications under the considered strategies and double-Rayleigh fading, (2) are unique as efforts to investigate PHY-security in CRVNs under the consideration of two spectrum sharing constraints and double-Rayleigh fading channels is made first time in the literature, and (3) lay the foundation for examining PHY-security in CRVNs over more generalized cascaded fading models, such as N*Rayleigh and N*Nakagami-*m*. Specifically, in this work, under the two considered power control strategies and by taking double-Rayleigh fading channels into account, we investigate SOP, asymptotic SOP behavior, and ESC, for the considered underlay CRVNs. The key contributions of the paper are summarized as follows.

1. We deduce the exact SOP expressions over double-Rayleigh fading channels in order to investigate the secrecy performance under two considered power control strategies. These SOP expressions enable us to effectively determine the impact of key system/channel parameters on the system’s secrecy performance.
2. We further present the asymptotic SOP expressions for Strategy I and Strategy II over double-Rayleigh fading channels. These asymptotic expressions provide us some important insights related to the system’s achievable secrecy diversity order. Based on these asymptotic results, it is observed that the system can achieve a secrecy diversity order of 1, when the average channel gain of the main link goes to infinity and the average channel gain of the wiretap link is fixed. However, the convergence of achieving the asymptotical secrecy diversity order of 1 is very slow, due the involvement of double-Rayleigh fading channels. In addition, the secrecy diversity order reduces to zero when the average channel gains of the main and wiretap links go to infinity.
3. Using the derived exact SOP expression under Strategy II, we demonstrate the impact of maximum tolerable interference level and maximum secondary transmitter power on the secrecy performance. Specifically, we analyze two cases, viz., (1) when maximum tolerable interference level is proportional to maximum secondary transmitter power, and (2) when maximum tolerable interference level is not related to maximum secondary transmitter power. It is revealed from these two cases that the SOP performance saturates when maximum secondary transmitter power is large enough, which results into a zero system’ secrecy diversity gain.
4. Further, we deduce novel ESC expressions for Strategy I and Strategy II under double-Rayleigh fading, in order to analyze the impact of interference threshold, maximum transmit power, and average channel gains on the secrecy performance. We also present two key observations irrespective of two power control strategies, when the average channel gains of main and wiretap links are very large, i.e., (1) there exists a ceiling of ESC, and (2) the ESC follows a scaling law of $\Theta \left(\ln\left(\frac{\Omega_{m}^{2}}{\Omega_{e}^{2}}\right)\right)$, where $\Omega_{m}$ and $\Omega_{e}$ are the average channel gains of main and wiretap links, respectively.
5. We finally verify our analytical and theoretical findings via simulation studies. Our results show the impact of involved network parameters on the system’s SOP and ESC performances under Strategy I and Strategy II.

### 1.4. Organization

The paper is structured as follows: Section 2 describes the considered system model for CRVNs. In Section 3, we analyze and present the exact and asymptotic SOP expressions under Strategy I and Strategy II for CRVNs. Section 4 presents the ESC expressions under Strategies I and II. Numerical results are provided in Section 5 to offer valuable insights onto the secrecy performance. Finally, Section 6 concludes the work.

*Notations:*$K_{v}\left(\cdot \right)$ is the *v*-th order modified Bessel function of second kind (eq. (8.432)) of [48], ${}_{2}{\tilde{F}}_{1}\left(m,n,p;z\right)$ is the Hypergeometric regularized function (eq. (9.10)) of [48], Ψ(·,·,·) being the Kummer hypergeometric function (eq. (9.238)) of [48], $G_{p,q}^{m,n}\left(y{|}_{b_{1},\cdots ,b_{q}}^{a_{1},\cdots ,a_{p}}\right)$ is the Meijer-*G* function (eq. (9.301)) of [48], and $G_{p_{1},q_{1}:p_{2},q_{2}:p_{3},q_{3}}^{m_{1},n_{1}:n_{2},m_{2}:n_{3},m_{3}}\left(y,z{|}_{b_{1},\cdots ,b_{q_{1}}}^{a_{1},\cdots ,a_{p_{1}}}{|}_{d_{1},\cdots ,d_{q_{2}}}^{c_{1},\cdots ,c_{p_{2}}}{|}_{f_{1},\cdots ,f_{q_{3}}}^{e_{1},\cdots ,e_{p_{3}}}\right)$ is the extended generalized bivariate Meijer-*G* function (eq. (07.34.21.0081.01)) of [49].

## 2. System and Channel Models

In the following subsections, we detail the adopted cascaded fading channel model for the V2V channels and the system model for our considered cognitive vehicular networks. Further, we consider two power control strategies to minimize the interference at the primary receiver and present the end-to-end instantaneous signal-to-noise ratios for both the strategies.

### 2.1. Statistical Background: The Double-Rayleigh Distribution

As proposed in [33], for mobile-to-mobile links, the multiple Rayleigh propagation considers two or more independent Rayleigh fading processes generated by independent groups of scatterers around the two moving vehicles. The resulting transfer function, H(t), can be expressed as a linear combination of components with Rayleigh, double-Rayleigh, triple-Rayleigh, etc., distributed amplitudes. For the case of only double-Rayleigh process, the narrow-band, base-band channel transfer function can be written as [37]

(1) $$\begin{array}{c} \;\;\;\;\bar{H}\left(t\right)\;=\;\sqrt{\;\frac{2}{N_{T}N_{R}}}\;\sum_{n=1}^{N_{T}}\;\sum_{m=1}^{N_{R}}\;\;e^{j2\pi (f_{T}\cos\left(\phi_{n}\right)t+f_{R}\cos\left(\phi_{m}\right)t+\theta_{nm})}, \end{array}$$

where $N_{T}$ and $N_{R}$ are the respective numbers of scatterers generated around moving transmitter and receiver, $f_{T}$ and $f_{R}$ denote the respective maximum Doppler shift due to the motion (speed of mobility) of transmitter and receiver, $\phi_{n}$ and $\phi_{m}$ are the random angle of departure and the angle of arrival with respect to the velocity vectors, respectively, and $\theta_{nm}$ is the joint phase shift. It is important to note that the motions (speed of mobility) of transmitter and receiver are involved in the form of Doppler shifts to determine how fast the fading channel will be. For mathematical tractability, the channel between moving vehicles is assumed to be quasi-stationary for a short duration (i.e., one fading block time), and the distance between the nodes is much greater than the scattering radii, the channel between those moving vehicles can be distributed as double-Rayleigh fading (It is a more realistic channel model in a V2V scenario, especially when (i) the vehicles are moving in high scattering environment, e.g., high traffic scenarios and (ii) all the vehicles are equipped with low elevation antennas. Such a fading assumption which can find its applicability for vehicular communication scenarios in rush-hour traffic is widely investigated for vehicular networks in the literature [35,36,37,38,39,40,42,43]) [33,34,35,36,37,38,39,40,42,43,44,45,50,51,52].

Under double-Rayleigh fading, the resulting envelope R can be expressed as the product of $R_{1}$ and $R_{2}$, i.e., $R=R_{1}R_{2}$, where $R_{1}$ and $R_{2}$ are independent Rayleigh fading processes with mean powers $\Omega_{1}$ and $\Omega_{2}$, respectively. Thus, the probability density function (PDF) and the cumulative distribution function (CDF) of R can be expressed as [37]

(2) $$\begin{array}{cc} \; & f_{R}\left(r\right)=\frac{r}{\Omega_{1}\Omega_{2}}K_{0}\left(\frac{r}{\sqrt{\Omega_{1}\Omega_{2}}}\right), \end{array}$$

(3) $$\begin{array}{cc} \; & F_{R}\left(r\right)=1-\frac{r}{\sqrt{\Omega_{1}\Omega_{2}}}K_{1}\left(\frac{r}{\sqrt{\Omega_{1}\Omega_{2}}}\right), \end{array}$$

respectively. Moreover, the PDF and CDF of the square to the envelope, i.e., ${\left|R\right|}^{2}$, can be represented, respectively, as

(4) $$\begin{array}{cc} \; & f_{{\left|R\right|}^{2}}\left(r\right)=\frac{2}{\Omega_{1}\Omega_{2}}K_{0}\left(2\sqrt{\frac{r}{\Omega_{1}\Omega_{2}}}\right), \end{array}$$

(5) $$\begin{array}{cc} \; & F_{{\left|R\right|}^{2}}\left(r\right)=1-2\sqrt{\frac{r}{\Omega_{1}\Omega_{2}}}K_{1}\left(2\sqrt{\frac{r}{\Omega_{1}\Omega_{2}}}\right). \end{array}$$

### 2.2. Cognitive Radio Vehicular System

We consider a secure CRVN, where primary user vehicle and secondary user vehicles share the same licensed spectrum band in a given propagation environment. In the secondary network, the secondary transmitter sends its message to secondary receiver in the presence of a primary receiver present in the primary network. Meanwhile, in the secondary network, a passive eavesdropper vehicle is able to intercept the information transmitted by the secondary transmitter. Under the passive eavesdropping scenario, the instantaneous CSI between secondary transmitter and eavesdropper is not available at the secondary transmitter. During the whole process, the secondary transmitter imposes an interference to the primary receiver. Note that we highlight a practical consideration of the passive eavesdropping scenario since, in practice, the passive eavesdropper is noncooperative and does not feedback its instantaneous CSI to the trusted nodes. The assumption of known statistical CSI of eavesdropper’s channel can be applied to the scenario where the eavesdropper is part of a system which in alternate time slots becomes an active trusted user in the system. As such, the instantaneous CSI of the eavesdropper can be available at the transmitter via a feedback channel for the time slot where it is being served. Therefore, from this information and assuming eavesdropper CSI does not change under the assumption of quasi-stationary channel for a short duration, statistical CSI of the passive eavesdropper can be available at the trusted node, for the time slots where it is not being served [17,18,19,20,21,22,23,24,25,26,27,28,29,30]. The detail of the interference constraints is discussed later in this section.

**Figure 1:**
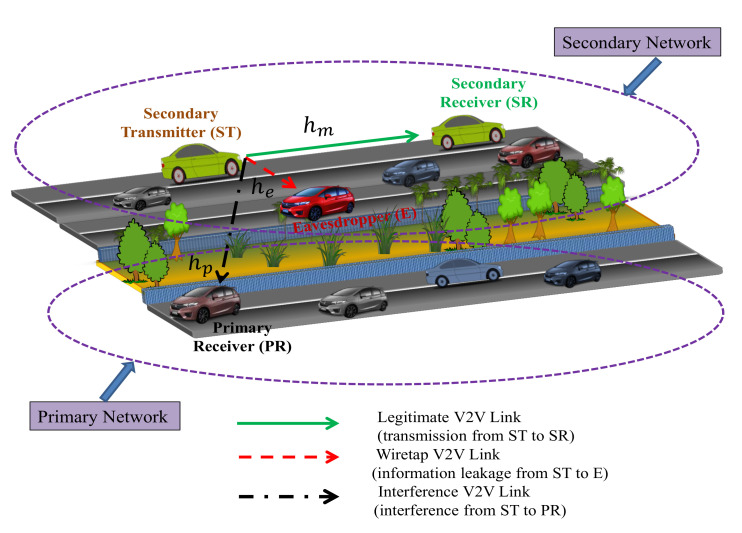
System model for the considered secure underlay CRVNs.

Figure 1 depicts the system model of the considered secure CRVN, which consists of a secondary transmitter vehicle ST, a secondary receiver vehicle SR, a primary receiver vehicle PR, and a passive eavesdropper vehicle E. All the terminals are equipped with a single antenna and operate in the half-duplex manner (note that the consideration of single-antenna at the terminals can reduce the system complexity and requirement of power-intensive signal processing modules and hence make them for practical use in various battery operated devices, such as wireless sensor applications. In addition, the assumption of half-duplex terminals can be practically applicable, as the half-duplex operation is much easier and does not require additional signal-processing operations compared to the full-duplex operation, because in the full-duplex operation, a significant amount of self-interference is observed at the receiving antenna as a result of the signal from the transmitting antenna of the same node). Since, all the nodes are moving vehicles; therefore, the channels for ST→SR (i.e., main link), ST→PR (i.e., interference link), and ST→E (i.e., wiretap link) links can be modeled as double-Rayleigh fading. We represent $h_{m}$, $h_{p}$, and $h_{e}$ as the channel coefficients of ST→SR, ST→PR, and ST→E links, respectively. In addition, we consider the perfect CSI knowledge of the channels. In this paper, we consider the perfect channel estimation process. However, imperfect channel estimates may be available for transmission in such systems, which are generally inaccurate and outdated with respect to actual channel. Consequently, the imperfect/outdated CSI for the actual channel can be expressed as $h_{ı}^{imperfect}=\varrho_{ı}h_{ı}+\sqrt{1-\varrho_{ı}^{2}}w_{ı}$, for ı∈{m,e,p}, where $\rho_{ı}$ denotes the normalized correlation coefficient between $h_{ı}^{imperfect}$ and $h_{ı}$, and $w_{ı}$ is a Gaussian random variable having the same variance as that of $h_{ı}$. Therefore, the performance evaluation of considered CRVNs under such imperfect/outdated CSI requires a fresh approach, which is studied thoroughly and comprehensively in the future work. Under double-Rayleigh fading, the channel coefficients $h_{ı}$, for ı∈{m,e,p}, can be expressed as the product of $h_{ı,1}$ and $h_{ı,2}$, where $h_{ı,1}$ and $h_{ı,2}$ are independent complex Gaussian random variables having zero mean and variance (without loss of generality, we assume $\Omega_{ı,1}=\Omega_{ı,2}=\Omega_{ı}$, for ı∈{m,e,p}; however, the analysis can readily be extended for $\Omega_{ı,1}\neq \Omega_{ı,2}$). $\Omega_{ı,1}$ and $\Omega_{ı,2}$, respectively. $P_{s}$ denotes the transmit power at ST. We also assume the additive white Gaussian noise (AWGN) with zero mean and $N_{0}$ variance for each link.

### 2.3. Instantaneous End-to-End Signal-to-Noise Ratio

Suppose that ST sends its confidential information to the legitimate SR over the main channel, and at the same time an E tries to decode this information through the wiretap channel; then, the signal received at SR and E can be given by $y_{\mathrm{SR}}=\sqrt{P_{s}}h_{m}x_{s}+n_{m}$ and $y_{E}=\sqrt{P_{s}}h_{e}x_{s}+n_{e}$, respectively, where $n_{m}$ and $n_{e}$ are AWGNs at SR and E, respectively. The instantaneous end-to-end signal-to-noise ratios (SNRs) $\Lambda_{m}$ and $\Lambda_{e}$ at SR and E can be expressed as

(6) $$\begin{array}{c} \Lambda_{m}=\frac{P_{s}{\left|h_{m}\right|}^{2}}{N_{0}}\;\mathrm{and}\;\Lambda_{e}=\frac{P_{s}{\left|h_{e}\right|}^{2}}{N_{0}}. \end{array}$$

Furthermore, we assume that PR feedbacks its instantaneous CSI to secondary transmitter ST, and ST accordingly adjusts its transmit power to satisfy the interference constraint [52]. Practically, a spectrum band manager can help to realize this task by mediating between the primary and the secondary users. Therefore, in order to protect the QoS of PR, we employ two power control strategies at ST, i.e., (1) Strategy I: single-power constraint of the interference on the PR, $I_{P}$ [22,28,53] and (2) Strategy II: combined power constraint of the interference on the PR and the maximum transmit power at ST, *Q* [29,30,53].

*Strategy I:* Under Strategy I, the transmit power $P_{s}$ at ST is constrained so that the interference impinged on PR remains below the maximum tolerable interference level $I_{P}$. Therefore, $P_{s}$ at ST can be mathematically expressed as $P_{s}=\frac{I_{P}}{|h_{p}{|}^{2}}$. Therefore, with Strategy I, the instantaneous end-to-end SNRs at SR and E can be given as

(7) $$\begin{array}{c} \Lambda_{m}^{I}=\frac{\rho |h_{m}{|}^{2}}{|h_{p}{|}^{2}}\;\mathrm{and}\;\Lambda_{e}^{I}=\frac{\rho |h_{e}{|}^{2}}{|h_{p}{|}^{2}}, \end{array}$$

respectively, where $\rho ≜\frac{I_{P}}{N_{0}}$.

*Strategy II:* In Strategy II, if ST is power limited terminal, then ST may transmit up to the maximum transmit power constraint of *Q*, and therefore $P_{s}$ at ST can be expressed as $P_{s}=\min\left(\frac{I_{P}}{|h_{p}{|}^{2}},Q\right)$. Taking such strategy into account, the instantaneous end-to-end SNRs at SR and E can be given by

(8) $$\begin{array}{cc} \; & \Lambda_{m}^{II}=\min\left(\frac{\rho }{|h_{p}{|}^{2}},\rho_{1}\right){\left|h_{m}\right|}^{2}, \end{array}$$

(9) $$\begin{array}{cc} \; & \Lambda_{e}^{II}=\min\left(\frac{\rho }{|h_{p}{|}^{2}},\rho_{1}\right){\left|h_{e}\right|}^{2}, \end{array}$$

respectively, where $\rho_{1}≜\frac{Q}{N_{0}}$.

Under the two adopted strategies, the capacities corresponding to main link (i.e., ST→SR) and wiretap link (i.e., ST→E) can be given by $C_{m}^{ȷ}=\log_{2}\left(1+\Lambda_{m}^{ȷ}\right)$ and $C_{e}^{ȷ}=\log_{2}\left(1+\Lambda_{e}^{ȷ}\right)$, where ȷ=I for Strategy I and ȷ=II for Strategy II. Moreover, the secrecy capacity of the wireless transmission can be given as $C_{sec}^{ȷ}=\max\left\{C_{m}^{ȷ}-C_{e}^{ȷ},0\right\}$, for ȷ∈{I,II}.

**Figure 2:**
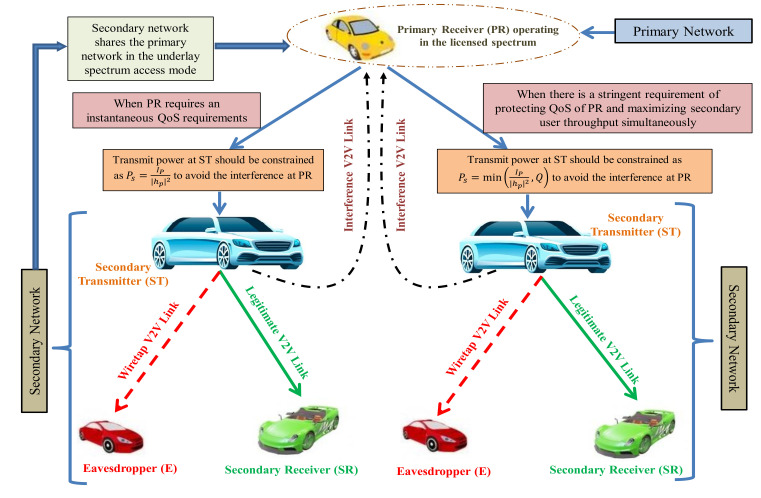
Overall representation of the considered CRVN framework.

In addition, Figure 2 shows the overall representation of the proposed CRVN framework. Here, the secondary vehicular network operates in the underlay spectrum sharing context along with the presence of a primary vehicular network. The transmission in the secondary network can only be established as long as the resulting interference on the PR is maintained underneath a given threshold. Firstly, if the PR generates an instantaneous QoS requirements, then the transmit power at ST should be constrained so that the interference imposed on PR remains below the maximum tolerable interference level, and consequently the transmit power, $P_{s}$, at ST can be given as $P_{s}=\frac{I_{P}}{|h_{p}{|}^{2}}$. Thereafter, the secondary users are allowed to use the licensed band and perform their operations accordingly. On the other hand, if the PR generates a stringent requirement of protecting QoS of PR and maintaining secondary user throughput simultaneously, then the transmit power, $P_{s}$, at ST can be expressed as $P_{s}=\min\left(\frac{I_{P}}{|h_{p}{|}^{2}},Q\right)$. Accordingly, the secondary users are allowed to start their transmissions. Finally, the performance of secure secondary network under the above two constraints can be evaluated in terms of SOP, asymptotic SOP, and ESC, as presented in subsequent Section 3 and Section 4.

### 2.4. Practical Applicability

The proposed analysis of the considered system by taking two power control strategies and double-Rayleigh fading channels into account can be applicable for various practical scenarios, as stated below.

- The proposed analysis under **Strategy I** can be more appropriate under the practical scenario when the service provided by the primary user has an instantaneous QoS requirement.
- The proposed analysis under **Strategy II** is suitable under the practical scenario when there is a stringent requirement of protecting QoS of primary user and maximizing secondary user throughput simultaneously.
- The proposed analysis under double-Rayleigh fading assumption is practically applicable for vehicular communication scenarios in rush-hour urban traffic.
- The proposed analysis is also applicable for the scenario when one mobile terminal is located indoors in low-ascent building, and the another mobile terminal is placed outdoors, as the cascaded fading envelope distribution is found suitable under such scenario.
- The proposed analysis can be applied to the scenario when the mobile nodes are located in a relatively dense scattering (e.g., vegetation) environment, as the channel between them will be a good fit for cascaded fading distribution.

It is to be noted that compared to the existing similar works presented in [44,45,47], the complexity of this work can be discussed as follows; (i) the paper [44] adopted N*Nakagami-*m* fading channels and [45] adopted cascaded fading channels, which implies that the cascading degree of order *N* imposes more computational resources in examining the performance of the considered system. Whereas this paper considers the double-Rayleigh fading channels, which allows one to operate with less computational resources (because of having cascading degree of order (2) while evaluating the system performance, without the loss of information, and (ii) the work presented in [47] considered the multiple-antennas at the legitimate destination and eavesdropper, which require several parallel radio frequency chains in the front-end architecture of the receiver. This increases the power consumption, complexity, cost, and size of the system, due to which the direct implementation of such systems is hindered in battery-operated sources, such as in wireless sensor applications. That said, this paper considers the single-antenna terminals which drastically reduce the system’s complexity and can be efficiently applicable for the resource constraint devices.

Furthermore, we evaluate the SOP and ESC for Strategy I and Strategy II under double-Rayleigh fading channels, in what follows. Note that SOP is an appropriate metric for the block fading channels (such as, double-Rayleigh fading channels under multiple scattering phenomenon for vehicular scenario), where the maximum rate of reliable communication is supported only by the one channel realization. On the other hand, ESC is the maximum mutual information averaged over many independent fades of the channel. With the block fading, the time average should converge to the same limit for almost all channel realizations of the fading process (known as ergodicity); thus, ESC is only the long-term time average rate achieved, and not on how fast that rate fluctuates over the time. Therefore, we can evaluate the SOP and ESC in the one system assumption.

## 3. Exact and Asymptotic SOP Analyses under Strategies I and II

In the consequent subsections, we derive an analytical expression for a key secrecy metric and SOP to quantify the considered network secrecy performance under both Strategies I and II. Further, to provide meaningful insights, we also provide asymptotic SOP analysis under both the scenarios for the considered system.

### 3.1. Strategy I: Single-Power Constraint of the Interference on the PR

#### 3.1.1. Exact Analysis for SOP

The SOP can be defined as the probability that the achievable secrecy capacity is less than a predefined secrecy transmission rate $R_{s}$ (in bps/Hz). We can mathematically express the SOP under Strategy I as

(10) $$\begin{array}{cc} P_{out}^{sec,I} & =\mathrm{Pr}[\max\left\{C_{m}^{I}-C_{e}^{I},0\right\}<R_{s}]. \end{array}$$

Note that when $C_{m}^{I}\leq C_{e}^{I}$, the secrecy is compromised, i.e., $P_{out}^{sec,I}=1$. Therefore, we analyze the SOP when $C_{m}^{I}>C_{e}^{I}$ as

(11) $$\begin{array}{cc} P_{out}^{sec,I} & =\mathrm{Pr}\left[C_{m}^{I}-C_{e}^{I}<R_{s}\right]=\mathrm{Pr}\left[\frac{1+\frac{\rho |h_{m}{|}^{2}}{|h_{p}{|}^{2}}}{1+\frac{\rho |h_{e}{|}^{2}}{|h_{p}{|}^{2}}}<\eta \right]\\ \; & =1-Pr\left[|h_{e}{|}^{2}<\frac{|h_{m}{|}^{2}}{\eta }-\frac{\left(\eta -1\right)|h_{p}{|}^{2}}{\eta \rho }\right]. \end{array}$$

Since $|h_{m}{|}^{2}$ and $|h_{e}{|}^{2}$ consist of a common channel gain $|h_{p}{|}^{2}$; therefore, the SOP under this strategy can be expressed as

(12) $$\begin{array}{cc} \; & P_{out}^{sec,I}=1-\int_{0}^{\infty }\left[\underset{≜I_{1}}{\underset{︸}{\int_{\frac{(\eta -1)w}{\rho }}^{\infty }F_{|h_{e}{|}^{2}}\left(\frac{y}{\eta }-\frac{(\eta -1)w}{\eta \rho }\right)f_{|h_{m}{|}^{2}}\left(y\right)dy}}\right]f_{|h_{p}{|}^{2}}\left(w\right)dw, \end{array}$$

where $\eta =2^{R_{s}}$ is the secrecy target threshold. To evaluate the SOP under Strategy I in (12), we first need to simplify the inner integral $I_{1}$, which is given as per the following theorem.

**Theorem** **1.** *The inner integral $I_{1}$ of (12) can be expressed as*

(13) $$\begin{array}{c} I_{1}=I_{1a}-I_{1b}, \end{array}$$

*where*

(14) $$\begin{array}{cc} I_{1a} & =2\sqrt{\frac{(\eta -1)w}{\rho \lambda_{m}}}K_{1}\left(2\sqrt{\frac{(\eta -1)w}{\rho \lambda_{m}}}\right), \end{array}$$

(15) $$\begin{array}{cc} I_{1b} & =\frac{\sqrt{\lambda_{m}}}{\sqrt{\eta \lambda_{e}}}\;\sum_{k=0}^{\infty }\frac{{\left(-1\right)}^{k}}{k!}{\left(\frac{\eta -1}{\rho \lambda_{m}}\right)}^{k}w^{k}G_{3,3}^{2,3}\left(\frac{\lambda_{m}}{\eta \lambda_{e}}{|}_{\frac{1}{2},-\frac{1}{2},k-\frac{1}{2}}^{-\frac{1}{2},k-\frac{1}{2},k-\frac{1}{2}}\right), \end{array}$$

*where $\lambda_{m}=\Omega_{m}^{2}$ and $\lambda_{e}=\Omega_{e}^{2}$.*

**Proof.**  The proof is given in Appendix A. □

Furthermore, invoking (14) and (15) along with the PDF of $|h_{p}{|}^{2}$ into (12), we can represent the SOP as

(16) $$\begin{array}{cc} \; & P_{out}^{sec,I}\left(\eta \right)=1-\frac{4}{\lambda_{p}}\sqrt{\frac{\left(\eta -1\right)}{\rho \lambda_{m}}}\left[\int_{0}^{\infty }w^{\frac{1}{2}}K_{0}\left(2\sqrt{\frac{w}{\lambda_{p}}}\right)K_{1}\left(2\sqrt{\frac{(\eta -1)w}{\rho \lambda_{m}}}\right)dw\right]\\ \; & \;+\;\frac{2\sqrt{\lambda_{m}}}{\lambda_{p}\sqrt{\eta \lambda_{e}}}\sum_{k=0}^{\infty }\frac{{\left(-1\right)}^{k}}{k!}{\left(\frac{\eta -1}{\rho \lambda_{m}}\right)}^{k}G_{3,3}^{2,3}\left(\frac{\lambda_{m}}{\eta \lambda_{e}}{|}_{\frac{1}{2},-\frac{1}{2},k-\frac{1}{2}}^{-\frac{1}{2},k-\frac{1}{2},k-\frac{1}{2}}\right)\left[\;\int_{0}^{\infty }\;\;\;w^{k}K_{0}\left(2\sqrt{\frac{w}{\lambda_{p}}}\right)dw\right], \end{array}$$

where $\lambda_{p}=\Omega_{p}^{2}$. Then, the first integral in (16) can be simplified using (eq. (03.04.26.0009.01)) of [49] and (eq. (07.34.21.0011.01)) of [49], and the second integral in (16) can be evaluated by first using the transformation of variables $\frac{w}{\lambda_{p}}=\frac{t^{2}}{4}$ and then applying (eq. (6.561.16)) of [48]. Consequently, the SOP under Strategy I, $P_{out}^{sec,I}\left(\eta \right)$, can be expressed as

(17) $$\begin{array}{cc} P_{out}^{sec,I}\left(\eta \right) & =1-\sqrt{\frac{\left(\eta -1\right)\lambda_{p}}{\rho \lambda_{m}}}G_{2,2}^{2,2}\left(\frac{\left(\eta -1\right)\lambda_{p}}{\rho \lambda_{m}}{|}_{\frac{1}{2},-\frac{1}{2}}^{-\frac{1}{2},-\frac{1}{2}}\right)\\ \; & +\frac{\sqrt{\lambda_{m}}}{\sqrt{\eta \lambda_{e}}}\sum_{k=0}^{\infty }\frac{{\left(-1\right)}^{k}}{k!}{\left(\frac{\eta -1}{\rho \lambda_{m}}\right)}^{k}\lambda_{p}^{k}{\left(\Gamma \left(k+1\right)\right)}^{2}G_{3,3}^{2,3}\left(\frac{\lambda_{m}}{\eta \lambda_{e}}{|}_{\frac{1}{2},-\frac{1}{2},k-\frac{1}{2}}^{-\frac{1}{2},k-\frac{1}{2},k-\frac{1}{2}}\right). \end{array}$$

**Remark** **1.** *The SOP in (17) mainly consists of powers, complete Gamma function, and Meijer-G functions, containing maximum interference threshold limit $\left(I_{p}\right)$, secrecy target threshold (η), and average channel gains ($\Omega_{m}$, $\Omega_{e}$, and $\Omega_{p}$), which can effectively be evaluated using Mathematica software. The SOP behavior for various values of channel/system parameters is shown numerically in Section 5.*

**Remark** **2.** *We infer that the SOP expression in (17) depends on the average channel gain of the interference link (ST → PR), i.e., $\lambda_{p}=\Omega_{p}^{2}$, which implies that the SOP performance degrades as $\lambda_{p}$ increases and vice versa. This is due to the fact that the power at ST reduces with the increased $\lambda_{p}$, as also validated numerically in Section 5.*

#### 3.1.2. Asymptotic Analysis for SOP

To gain more insights into the achievable secrecy diversity order of the considered system, we focus on the asymptotic analysis in the high average channel fading gains regime. Here, we specifically investigated two separate scenarios: (1) when $\lambda_{m}\to \infty$ and $\lambda_{e}$ is fixed. In this scenario, the quality of the legitimated channel is better than the quality of wiretap channel (i.e., E is located far away from ST), and (2) when $\lambda_{m}\to \infty$ and $\lambda_{e}\to \infty$, where both the legitimated and wiretap channels experience similar fading conditions. Note that there may be another scenario where $\lambda_{m}$ is fixed and $\lambda_{e}\to \infty$. However, this case significantly strengthens the quality of wiretap link and increases the probability of successful eavesdropping, as E, which implies that the secrecy diversity order becomes zero.

When $\lambda_{m}\to \infty$ and fixed $\lambda_{e}$

Under this scenario, we simplify (17) by ignoring the higher order infinitesimal terms to obtain the asymptotic SOP as

(18) $$\begin{array}{cc} P_{out,asy}^{sec,I}\left(\eta \right)\; & \underset{\lambda_{m}\to \infty }{≃}\;\;\;1-\sqrt{\frac{\left(\eta -1\right)\lambda_{p}}{\rho \lambda_{m}}}G_{2,2}^{2,2}\left(\frac{\left(\eta -1\right)\lambda_{p}}{\rho \lambda_{m}}{|}_{\frac{1}{2},-\frac{1}{2}}^{-\frac{1}{2},-\frac{1}{2}}\right)\\ \; & +\frac{\sqrt{\lambda_{m}}}{\sqrt{\eta \lambda_{e}}}G_{3,3}^{2,3}\left(\frac{\lambda_{m}}{\eta \lambda_{e}}{|}_{\frac{1}{2},-\frac{1}{2},-\frac{1}{2}}^{-\frac{1}{2},-\frac{1}{2},-\frac{1}{2}}\right). \end{array}$$

**Remark** **3.** *Secrecy diversity order analysis: When $\lambda_{m}\to \infty$ and $\lambda_{e}$ are fixed, the secrecy diversity order can be defined as the ratio of asymptotic SOP to average channel gain of the main link $\lambda_{m}$, yielding*

(19) $$\begin{array}{c} G_{D}=-\lim_{\lambda_{m}\to \infty }\frac{\log P_{out,asy}^{sec,I}\left(\eta \right)}{\log\lambda_{m}}. \end{array}$$

*From (18), we can observe that the term $G_{3,3}^{2,3}\left(\frac{\lambda_{m}}{\eta \lambda_{e}}{|}_{\frac{1}{2},-\frac{1}{2},-\frac{1}{2}}^{-\frac{1}{2},-\frac{1}{2},-\frac{1}{2}}\right)$ converges to zero very quickly as $\lambda_{m}\to \infty$ for fixed η and $\lambda_{e}$, and hence, it can be ignored from (18) while evaluating the secrecy diversity order. Consequently, we can re-express the resultant asymptotic SOP via (eq. (07.34.03.0871.01)) of [49], and after some simplifications, as*

(20) $$\begin{array}{c} P_{out,asy}^{sec,I}\left(\eta \right)≃1-\frac{A(\eta )}{\lambda_{m}}\;{}_{2}{\tilde{F}}_{1}\left(2,2,3;1-\frac{A(\eta )}{\lambda_{m}}\right), \end{array}$$

*where $A\left(\eta \right)=\frac{\left(\eta -1\right)\lambda_{p}}{\rho }$. Now, invoking (20) into (19), and by simplifying ${}_{2}{\tilde{F}}_{1}\left(a,b,c;z\right)$ using (eq. (07.24.26.0003.01)) of [49] and (eq. (07.23.03.3573.01)) of [49], and after some involved simplifications, we can express the secrecy diversity order as*

(21) $$\begin{array}{cc} \;G_{D}\; & =\;-\;\;\lim_{\lambda_{m}\to \infty }\;\;\frac{\log\left[1-\frac{\lambda_{m}\left(-A\left(\eta \right)+\lambda_{m}+A\left(\eta \right)\log\left(\frac{A(\eta )}{\lambda_{m}}\right)\right)}{{\left(A\left(\eta \right)-\lambda_{m}\right)}^{2}}\right]}{\log\lambda_{m}}, \end{array}$$

*which can be further simplified with the assistance of L’Hospital’s rule to obtain the secrecy diversity order as*

(22) $$\begin{array}{c} G_{D}=1. \end{array}$$

*Therefore, we can infer that the system can achieve a secrecy diversity order of 1 and does not depend on the parameters related to the wiretap link (i.e., ST → E) and interference link (i.e., ST → PR).*

**Figure 3:**
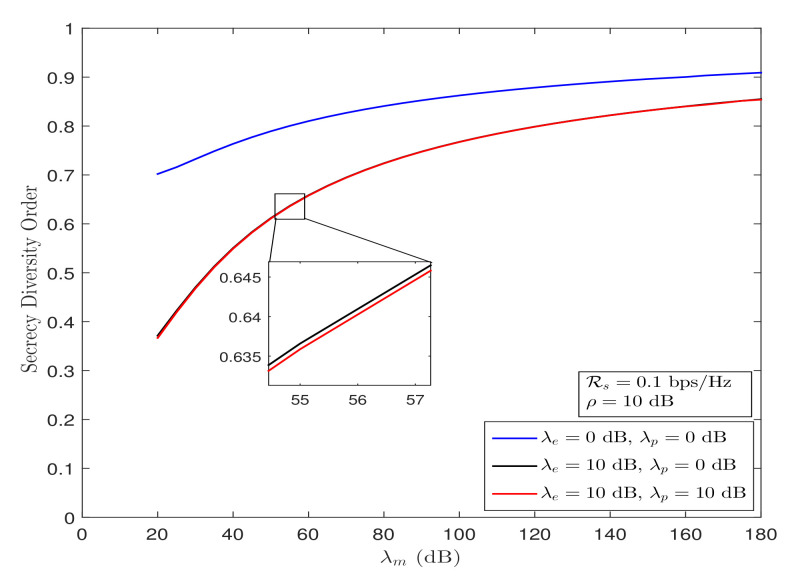
Secrecy diversity order behavior of the considered system under Strategy I for $\lambda_{m}\to \infty$ and fixed $\lambda_{e}$.

**Remark** **4.** *The convergence behavior of secrecy diversity order is shown in Figure 3, from which it can be observed that the secrecy diversity order converges to its asymptotical value of 1 over double-Rayleigh fading channels, irrespective of the wiretap link strength $\lambda_{e}$, ρ, and $\lambda_{p}$. However, the convergence gets slower because of the involved double-Rayleigh fading channels. We can also infer that the convergence further slows down as $\lambda_{e}$ and/or $\lambda_{p}$ increases and vice versa.*

When $\lambda_{m}\to \infty$ and $\lambda_{e}\to \infty$

The asymptotic SOP for this case can be evaluated as per the following theorem.

**Theorem** **2.** *The asymptotic SOP for the case when $\lambda_{m}\to \infty$ and $\lambda_{e}\to \infty$ (as the average channel gains of both the legitimated link and wiretap link are improved simultaneously) under double-Rayleigh fading channels can be expressed as*

(23) $$\begin{array}{c} P_{out,asy}^{sec,I}\left(\eta \right)\underset{\lambda_{m},\lambda_{e}\to \infty }{≃}1-\frac{\sqrt{\eta \lambda_{e}}}{\sqrt{\lambda_{m}}}G_{2,2}^{2,2}\left(\frac{\eta \lambda_{e}}{\lambda_{m}}{|}_{\frac{1}{2},-\frac{1}{2}}^{-\frac{1}{2},-\frac{1}{2}}\right). \end{array}$$

**Proof.**  Under $\lambda_{m}\to \infty$ and $\lambda_{e}\to \infty$, we can approximate the SOP as $P_{out}^{sec,I}\left(\eta \right)=Pr\left[\frac{1+\frac{\rho |h_{m}{|}^{2}}{|h_{p}{|}^{2}}}{1+\frac{\rho |h_{e}{|}^{2}}{|h_{p}{|}^{2}}}<\eta \right]\approx Pr\left[\frac{|h_{m}{|}^{2}}{|h_{e}{|}^{2}}<\eta \right]$, which can be further expressed in the integral form as $P_{out,asy}^{sec,I}\left(\eta \right)\approx \int_{0}^{\infty }F_{|h_{m}{|}^{2}}\left(\eta y\right)f_{|h_{e}{|}^{2}}\left(y\right)dy$. Now, invoking the CDF of $|h_{m}{|}^{2}$ and the PDF of $|h_{e}{|}^{2}$ and simplifying with the aid of (eq. (07.34.21.0011.01)) of [49], we can obtain the asymptotic SOP expression, as given in (23). □

**Remark** **5.** *From (23), we can infer that the secrecy outage floor occurs for fixed ratio $\frac{\lambda_{e}}{\lambda_{m}}$ (as $\lambda_{m}\to \infty$ and $\lambda_{e}\to \infty$), and hence, the secrecy diversity order cannot be attained. In addition, it is also worthwhile to note that the system’s secrecy diversity order can also be realized by analyzing the asymptotic SOP behavior for the case when $\rho =\frac{I_{p}}{N_{0}}\to \infty$. Under this case, we can have the same asymptotic SOP expression as evaluated in (23), since ρ at both D and E are increased simultaneously. We can further reveal that the SOP expression under this case achieves an error floor and results in a zero secrecy diversity order.*

### 3.2. Strategy II: Combined Power Constraint of the Interference at the PR and Maximum Transmit Power at the ST

#### 3.2.1. Exact Analysis for SOP

Considering $C_{m}^{II}>C_{e}^{II}$ and using (8) and (9), the SOP can be expressed as

(24) $$\begin{array}{cc} \; & P_{out}^{sec,II}\left(\eta \right)=\mathrm{Pr}\left[\frac{1+\min\left(\frac{\rho }{|h_{p}{|}^{2}},\rho_{1}\right){\left|h_{m}\right|}^{2}}{1+\min\left(\frac{\rho }{|h_{p}{|}^{2}},\rho_{1}\right){\left|h_{e}\right|}^{2}}<\eta \right]\\ \; & =\underset{≜\;\Theta_{1}\left(\eta \right)}{\underset{︸}{\mathrm{Pr}\left[\frac{1+\rho_{1}{\left|h_{m}\right|}^{2}}{1+\rho_{1}{\left|h_{e}\right|}^{2}}<\eta \right]\mathrm{Pr}\left[\frac{\rho }{\rho_{1}}\geq {\left|h_{p}\right|}^{2}\right]}}+\underset{≜\;\Theta_{2}\left(\eta \right)}{\underset{︸}{Pr\left[\frac{1+\frac{\rho |h_{m}{|}^{2}}{|h_{p}{|}^{2}}}{1+\frac{\rho |h_{e}{|}^{2}}{|h_{p}{|}^{2}}}<\eta ,\frac{\rho }{\rho_{1}}<{\left|h_{p}\right|}^{2}\right]}}. \end{array}$$

Further, the SOP in (24) can be simplified as per Theorem 3.

**Theorem** **3.** *The exact expression for the SOP under Strategy II using (24) can be expressed as*

(25) $$\begin{array}{c} P_{out}^{sec,II}\left(\eta \right)=\Theta_{1}\left(\eta \right)+\Theta_{2}\left(\eta \right), \end{array}$$

*where*

(26) $$\begin{array}{cc} \; & \Theta_{1}\left(\eta \right)=[1-2\sqrt{\frac{\eta -1}{\rho_{1}\lambda_{m}}}K_{1}\left(2\sqrt{\frac{\eta -1}{\rho_{1}\lambda_{m}}}\right)+\frac{\sqrt{\lambda_{m}}}{\sqrt{\eta \lambda_{e}}}\sum_{k=0}^{\infty }\frac{{\left(-1\right)}^{k}}{k!}{\left(\frac{\eta -1}{\rho_{1}\lambda_{m}}\right)}^{k}\\ \; & \times G_{3,3}^{2,3}\left(\frac{\lambda_{m}}{\eta \lambda_{e}}{|}_{\frac{1}{2},-\frac{1}{2},k-\frac{1}{2}}^{-\frac{1}{2},k-\frac{1}{2},k-\frac{1}{2}}\right)]\left[1-\frac{2\sqrt{\rho }}{\sqrt{\rho_{1}\lambda_{p}}}K_{1}\left(\frac{2\sqrt{\rho }}{\sqrt{\rho_{1}\lambda_{p}}}\right)\right], \end{array}$$

(27) $$\begin{array}{cc} \; & \Theta_{2}\left(\eta \right)=\frac{2\sqrt{\rho }}{\sqrt{\rho_{1}\lambda_{p}}}K_{1}\left(\frac{2\sqrt{\rho }}{\sqrt{\rho_{1}\lambda_{p}}}\right)-\frac{4}{\lambda_{p}}\sqrt{\frac{\eta -1}{\rho \lambda_{m}}}[\frac{\lambda_{p}^{\frac{3}{2}}}{4}\;G_{2,2}^{2,2}\left(\frac{\left(\eta -1\right)\lambda_{p}}{\rho \lambda_{m}}{|}_{\frac{1}{2},-\frac{1}{2}}^{-\frac{1}{2},-\frac{1}{2}}\right)\\ \; & -\frac{\rho^{\frac{3}{2}}}{2\rho_{1}^{\frac{3}{2}}}\sum_{i=1}^{N}g_{i}t_{i}^{2}G_{0,2}^{2,0}\left(\frac{\left(\eta -1\right)t_{i}^{2}}{\rho_{1}\lambda_{m}}|\frac{1}{2},-\frac{1}{2}\right)G_{0,2}^{2,0}\left(\frac{\rho t_{i}^{2}}{\rho_{1}\lambda_{p}}|0,0\right)]+\frac{\sqrt{\lambda_{m}}}{\sqrt{\eta \lambda_{e}}\lambda_{p}}\sum_{k=0}^{\infty }\frac{{\left(-1\right)}^{k}}{k!}\\ \; & \times {\left(\frac{\rho }{\rho_{1}}\right)}^{k+1}{\left(\frac{\eta -1}{\rho \lambda_{m}}\right)}^{k}G_{1,3}^{3,0}\left(\frac{\rho }{\rho_{1}\lambda_{p}}{|}_{-k-1,0,0}^{-k}\right)G_{3,3}^{2,3}\left(\frac{\lambda_{m}}{\eta \lambda_{e}}{|}_{\frac{1}{2},-\frac{1}{2},k-\frac{1}{2}}^{-\frac{1}{2},k-\frac{1}{2},k-\frac{1}{2}}\right), \end{array}$$

*where $g_{i}={\left\{\sum_{j=0}^{N-1}{\left[q_{j}\left(t_{i}\right)\right]}^{2}\right\}}^{-1}$ and $t_{i}$, (i=1,⋯,N) are the weights and zeros of N−order Gauss–Lobatto’s polynomial (eq. (25.4.33)) of [54], respectively, and $q_{N}\left(t\right)=\sqrt{2N+3}P_{N}^{\left(2,0\right)}\left(1-2t\right)$ with $P_{N}^{\left(2,0\right)}$ as the Jacobi polynomial.*

**Proof.**  The detailed analysis is given in Appendix B. □

**Remark** **6.** *We highlight that (25) mainly involves powers, Meijer-G functions, and modified Bessel function of the second kind, consisting of network parameters $I_{p}$, Q, η, $\Omega_{m}$, $\Omega_{e}$, and $\Omega_{p}$, which can readily be evaluated by the help of Mathematica software, as shown via numerical results in Section 5.*

**Remark** **7.** *The SOP expression in (25) consists of Gauss–Lobatto’s series expansion of order N, which converges to an arbitrarily accurate approximation by selecting the appropriate value of N. For instance, consider the term of (27), i.e., $Z=G_{0,2}^{2,0}\left(\frac{\left(\eta -1\right)t_{i}^{2}}{\rho_{1}\lambda_{m}}|\frac{1}{2},-\frac{1}{2}\right)G_{0,2}^{2,0}\left(\frac{\rho t_{i}^{2}}{\rho_{1}\lambda_{p}}|0,0\right)$. Note that the Meijer-G function can be expressed in terms of v-th order modified Bessel function of second kind using the transformation $K_{v}\left(x\right)=\frac{1}{2}G_{0,2}^{2,0}\left(\frac{x^{2}}{4}|\frac{v}{2},-\frac{v}{2}\right)$ (eq. (03.04.26.0009.01)) of [49], and $K_{v}\left(x\right)$ can further be expressed as $\sqrt{\pi }e^{-x}{\left(2x\right)}^{v}\Psi \left(v+0.5,1+2v;2x\right)$ (eq. (9.328)) of [48]. Realizing such representations in Z, we can get $Z=16\pi \sqrt{\frac{\left(\eta -1\right)t_{i}^{2}}{\rho_{1}\lambda_{m}}}e^{-2\sqrt{\frac{\left(\eta -1\right)t_{i}^{2}}{\rho_{1}\lambda_{m}}}}e^{-2\sqrt{\frac{\rho t_{i}^{2}}{\rho_{1}\lambda_{p}}}}\Psi \left(\frac{1}{2},1;4\sqrt{\frac{\rho t_{i}^{2}}{\rho_{1}\lambda_{p}}}\right)$ $\times \Psi \left(\frac{3}{2},3;4\sqrt{\frac{\left(\eta -1\right)t_{i}^{2}}{\rho_{1}\lambda_{m}}}\right)$. From which, it can be clearly seen that the exponential terms in Z implies that (27) decreases rapidly as N increases, and only a few values of N are sufficient to obtain satisfactory accuracy, as also shown numerically in Section 5.*

#### 3.2.2. Asymptotic Analysis for SOP

We analyze the asymptotic SOP performance of the considered system under Strategy II for two separate scenarios, i.e., (1) when $\lambda_{m}\to \infty$ and $\lambda_{e}$ is fixed and (2) when $\lambda_{m}\to \infty$ and $\lambda_{e}\to \infty$, in what follows.

When $\lambda_{m}\to \infty$ and fixed $\lambda_{e}$

For $\lambda_{m}\to \infty$ and fixed $\lambda_{e}$, by neglecting the higher order infinitesimal terms in (26) and (27), and then invoking the resultant expressions on (25), the asymptotic SOP expression can be given as

(28) $$\begin{array}{cc} \; & P_{out,asy}^{sec,II}\left(\eta \right)≃\left[1-2\sqrt{\frac{\eta -1}{\rho_{1}\lambda_{m}}}K_{1}\left(2\sqrt{\frac{\eta -1}{\rho_{1}\lambda_{m}}}\right)+\frac{\sqrt{\lambda_{m}}}{\sqrt{\eta \lambda_{e}}}G_{3,3}^{2,3}\left(\frac{\lambda_{m}}{\eta \lambda_{e}}{|}_{\frac{1}{2},-\frac{1}{2},-\frac{1}{2}}^{-\frac{1}{2},-\frac{1}{2},-\frac{1}{2}}\right)\right]\\ \; & \times \left[1-\frac{2\sqrt{\rho }}{\sqrt{\rho_{1}\lambda_{p}}}K_{1}\left(\frac{2\sqrt{\rho }}{\sqrt{\rho_{1}\lambda_{p}}}\right)\right]+\frac{2\sqrt{\rho }}{\sqrt{\rho_{1}\lambda_{p}}}K_{1}\left(\frac{2\sqrt{\rho }}{\sqrt{\rho_{1}\lambda_{p}}}\right)-\sqrt{\frac{\left(\eta -1\right)\lambda_{p}}{\rho \lambda_{m}}}\\ \; & \times G_{2,2}^{2,2}\left(\frac{\left(\eta -1\right)\lambda_{p}}{\rho \lambda_{m}}{|}_{\frac{1}{2},-\frac{1}{2}}^{-\frac{1}{2},-\frac{1}{2}}\right)-\frac{2\rho }{\rho_{1}^{\frac{3}{2}}\lambda_{p}}\sqrt{\frac{\left(\eta -1\right)}{\lambda_{m}}}\sum_{i=1}^{N}g_{i}t_{i}^{2}G_{0,2}^{2,0}\left(\frac{\left(\eta -1\right)t_{i}^{2}}{\rho_{1}\lambda_{m}}|\frac{1}{2},-\frac{1}{2}\right)\\ \; & \times G_{0,2}^{2,0}\left(\frac{\rho t_{i}^{2}}{\rho_{1}\lambda_{p}}|0,0\right)+\frac{\sqrt{\lambda_{m}}}{\sqrt{\eta \lambda_{e}}\lambda_{p}}G_{1,3}^{3,0}\left(\frac{\rho }{\rho_{1}\lambda_{p}}{|}_{-k-1,0,0}^{-k}\right)G_{3,3}^{2,3}\left(\frac{\lambda_{m}}{\eta \lambda_{e}}{|}_{\frac{1}{2},-\frac{1}{2},-\frac{1}{2}}^{-\frac{1}{2},-\frac{1}{2},-\frac{1}{2}}\right). \end{array}$$

**Figure 4:**
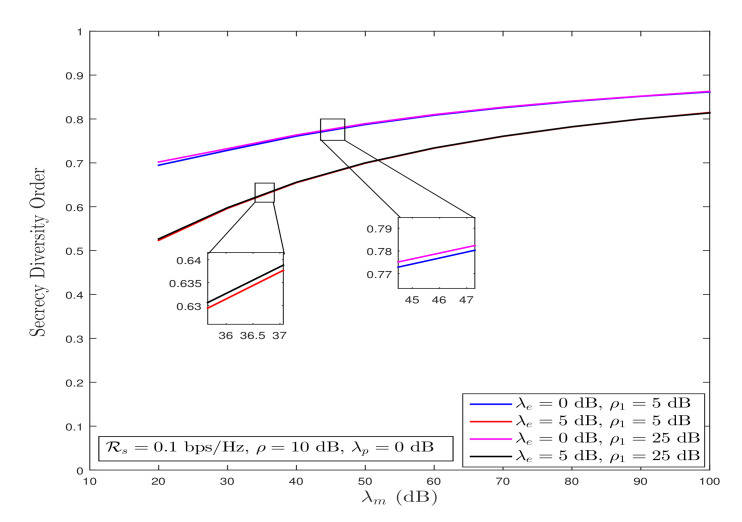
System’s secrecy diversity order behavior under Strategy II for $\lambda_{m}\to \infty$ and fixed $\lambda_{e}$.

**Remark** **8.** *By following a similar approach used to evaluate (22), we can infer from (28) that the secrecy diversity order of 1 can also be achieved under Strategy II. Furthermore, Figure 4 shows that the secrecy diversity order convergence slows down because of the involvement of double-Rayleigh fading channels, for various values of $\lambda_{e}$ and $\rho_{1}$.*

When $\lambda_{m}\to \infty$ and $\lambda_{e}\to \infty$

Using (24), the asymptotic SOP can be expressed as

(29) $$\begin{array}{cc} P_{out,asy}^{sec,II}\left(\eta \right) & \underset{\lambda_{m},\lambda_{e}\to \infty }{≃}\mathrm{Pr}\left[\frac{|h_{m}{|}^{2}}{|h_{e}{|}^{2}}<\eta \right]\left(Pr\left[|h_{p}{|}^{2}\leq \frac{\rho }{\rho_{1}}\right]+Pr\left[|h_{p}{|}^{2}>\frac{\rho }{\rho_{1}}\right]\right)\\ \; & \;\;=\int_{0}^{\infty }F_{|h_{m}{|}^{2}}\left(\eta y\right)f_{|h_{e}{|}^{2}}\left(y\right)dy. \end{array}$$

Now, invoking the CDF of $|h_{m}{|}^{2}$ and the PDF of $|h_{e}{|}^{2}$ into (29), and simplifying it via (eq. (07.34.21.0011.01)) of [49], we can obtain the asymptotic SOP expression under the scenario when $\lambda_{m}\to \infty$ and $\lambda_{e}\to \infty$ as

(30) $$\begin{array}{c} P_{out,asy}^{sec,II}\left(\eta \right)\underset{\lambda_{m},\lambda_{e}\to \infty }{≃}1-\frac{\sqrt{\eta \lambda_{e}}}{\sqrt{\lambda_{m}}}G_{2,2}^{2,2}\left(\frac{\eta \lambda_{e}}{\lambda_{m}}{|}_{\frac{1}{2},-\frac{1}{2}}^{-\frac{1}{2},-\frac{1}{2}}\right). \end{array}$$

**Remark** **9.** *According to (30), the asymptotic SOP in this scenario depends on the wiretap channel gain to the legitimated channel gain ratio, i.e., $\frac{\lambda_{e}}{\lambda_{m}}$. Therefore, we can infer that the secrecy outage floor occurs, which yields into a zero secrecy diversity order.*

#### 3.2.3. Impact of Maximum Tolerable Interference Level $I_{P}$ and Maximum Secondary Transmitter Power *Q*

It can be observed in (25) that the SOP expression under Strategy II also depends on the maximum tolerable interference level $I_{P}$ and maximum secondary transmitter power *Q*. Therefore, in order to study the impact of $I_{P}$ and *Q* on the system’s secrecy diversity gain, two cases, i.e., Case 1: when $\rho =\mu \rho_{1}$ and Case 2: when $\rho \neq \mu \rho_{1}$, where $\rho ≜\frac{I_{P}}{N_{0}}$ and $\rho_{1}≜\frac{Q}{N_{0}}$ are investigated in the following.

Case 1 $\left(\rho =\mu \rho_{1}\right)$

When ρ is proportional to $\rho_{1}$, i.e., $\rho =\mu \rho_{1}$, where μ is a positive constant. In the high SNR regime, i.e., $\rho_{1}\to \infty$, the SOP in (25) can be approximated by applying the fact $K_{1}\left(x\right)\underset{x\to 0}{\approx }\frac{1}{x}$ (eq. (9.6.9)) of [54] and ignoring the higher order infinitesimal terms at high SNR, as

(31) $$\begin{array}{cc} \; & P_{out}^{sec,II}\left(\eta \right)\underset{\rho =\mu \rho_{1},\rho_{1}\to \infty }{\approx }\frac{\sqrt{\lambda_{m}}}{\sqrt{\eta \lambda_{e}}}G_{3,3}^{2,3}\left(\frac{\lambda_{m}}{\eta \lambda_{e}}{|}_{\frac{1}{2},-\frac{1}{2},-\frac{1}{2}}^{-\frac{1}{2},-\frac{1}{2},-\frac{1}{2}}\right)\left(1-\frac{2\sqrt{\rho }}{\sqrt{\lambda_{p}\rho_{1}}}K_{1}\left(\frac{2\sqrt{\rho }}{\sqrt{\lambda_{p}\rho_{1}}}\right)\right)+\frac{2\sqrt{\rho }}{\sqrt{\lambda_{p}\rho_{1}}}\\ \; & \times K_{1}\left(\frac{2\sqrt{\rho }}{\sqrt{\lambda_{p}\rho_{1}}}\right)frac\sqrt{\lambda_{m}}\rho \sqrt{\eta \lambda_{e}}\lambda_{p}\rho_{1}G_{1,3}^{3,0}\left(\frac{\rho }{\rho_{1}\lambda_{p}}{|}_{-1,0,0}^{0}\right)G_{3,3}^{2,3}\left(\;\frac{\lambda_{m}}{\eta \lambda_{e}}{|}_{\frac{1}{2},-\frac{1}{2},-\frac{1}{2}}^{-\frac{1}{2},-\frac{1}{2},-\frac{1}{2}}\right). \end{array}$$

**Remark** **10.** *We can see from (31) that the SOP is independent of SNR $\rho_{1}$ with fixed ratio $\frac{\rho }{\rho_{1}}$, which implies that the secrecy diversity gain cannot be achieved in this case.*

Case 2 $\left(\rho \neq \mu \rho_{1}\right)$

When $\rho \neq \mu \rho_{1}$ and ρ is a constant. At high SNR range, i.e., $\rho_{1}\to \infty$, we can approximate the SOP expression in (25) by using the fact $K_{1}\left(x\right)\underset{x\to 0}{\approx }\frac{1}{x}$ (eq. (9.6.9)) of [54] and eliminating the higher order terms under high SNR $\left(\rho_{1}\to \infty \right)$ regime, as

(32) $$\begin{array}{cc} \; & \;\;\;P_{out}^{sec,II}\left(\eta \right)\\ \; & \;\;\;\underset{\rho \neq \mu \rho_{1},\rho_{1}\to \infty }{\approx }\;\;1-\frac{\sqrt{\left(\eta -1\right)\lambda_{p}}}{\sqrt{\rho \lambda_{m}}}G_{2,2}^{2,2}\left(\;\frac{\left(\eta -1\right)\lambda_{p}}{\rho \lambda_{m}}{|}_{\frac{1}{2},-\frac{1}{2}}^{-\frac{1}{2},-\frac{1}{2}}\right). \end{array}$$

**Remark** **11.** *From (32), it is noted that the SOP only depends on a constant ρ, although $\rho_{1}\to \infty$. This implies that the secrecy diversity gain reduces to zero in this case as well.*

## 4. ESC Analysis under Strategies I and II

### 4.1. Strategy I: Single-Power Constraint of the Interference on the PR

The instantaneous secrecy capacity for the considered secure CRVN under Strategy I can be given as

(33) $$\begin{array}{cc} C_{sec}^{I} & =C_{m}^{I}-C_{e}^{I}\\ \; & =\log_{2}\left(1+\Lambda_{m}^{I}\right)-\log_{2}\left(1+\Lambda_{e}^{I}\right). \end{array}$$

By averaging the instantaneous secrecy capacity expression over the distributions of the end-to-end SNRs $\Lambda_{m}^{I}$ and $\Lambda_{e}^{I}$ under Strategy I, the ESC can be expressed as

(34) $$\begin{array}{cc} \; & {\bar{C}}_{sec}^{I}=E\left[\log_{2}\left(1+\Lambda_{m}^{I}\right)-\log_{2}\left(1+\Lambda_{e}^{I}\right)\right],\\ \; & =\frac{1}{\ln(2)}E\left[\log\left(1\;+\frac{\rho |h_{m}{|}^{2}}{|h_{p}{|}^{2}}\;\right)\;-\log\left(1+\frac{\rho |h_{e}{|}^{2}}{|h_{p}{|}^{2}}\right)\right], \end{array}$$

which can be evaluated as per the following theorem.

**Theorem** **4.** *The exact ESC expression for the considered system under Strategy I over double-Rayleigh fading channels can be represented as*

(35) $$\begin{array}{cc} {\bar{C}}_{sec}^{I} & =\frac{1}{\ln(2)}\sum_{i=1}^{U}w_{i}e^{t_{i}}[\frac{t_{i}}{\rho \lambda_{m}}G_{2,4}^{4,1}\left(\frac{t_{i}}{\rho \lambda_{m}}{|}_{0,0,-1,-1}^{-1,0}\right)\\ \; & -t_{i}^{\frac{3}{2}}\frac{\sqrt{\lambda_{m}}+\sqrt{\lambda_{e}}}{\rho^{\frac{3}{2}}\lambda_{m}\lambda_{e}}S\left(\frac{t_{i}}{\rho }\right)]\frac{1}{\lambda_{p}}G_{0,2}^{2,0}\left(\frac{t_{i}}{\lambda_{p}}|0,0\right), \end{array}$$

*where $S\left(a\right)=G_{2,2:0,2:0,2}^{2,1:2,0:2,0}\left({}_{-\frac{3}{2},-\frac{3}{2}}^{-\frac{3}{2},-\frac{1}{2}}|0,0|\frac{1}{2},-\frac{1}{2}|\frac{a}{\lambda_{m}},\frac{a}{\lambda_{e}}\right)$. $w_{i}=\frac{t_{i}}{{\left(\left(U+1\right)L_{U+1}\left(t_{i}\right)\right)}^{2}}$ and $t_{i}$, (i=1,⋯,U) are the weights and zeros of U−order Gauss–Laguerre polynomial (i.e., $L_{U}\left(t\right)$) (eq. (25.5.45)) of [54].*

**Proof.**  See Appendix C for the proof. □

**Remark** **12.** *It can be seen from (35) that the ESC expression consists of exponential, powers, and Meijer-G function, involving system parameters $I_{p}$, η, $\Omega_{m}$, $\Omega_{e}$, and $\Omega_{p}$, and as such, it can be readily evaluated. In addition, the ESC expression in (35) consists of extended generalized bivariate Meijer-G function, which is not easily available in the Mathematica software computational package, but the work in [55] has proposed an efficient and accurate implementation in Mathematica. Moreover, from (35), we can see that the ESC expression consists of Gauss–Laguerre series expansion, which is convergent. We can achieve the accurate results by appropriately selecting the value of U (can be analytically proved as in Gauss–Lobatto’s polynomial in Remark 7), as also shown numerically in Section 5.*

**Remark** **13.** *Using (34) for $|h_{m}{|}^{2}\geq {\left|h_{e}\right|}^{2}$, the ESC under strategy I can be expressed as*

(36) $$\begin{array}{cc} {\bar{C}}_{sec}^{I} & =\frac{1}{\ln(2)}\int_{0}^{\infty }f_{|h_{e}{|}^{2}}\left(\right|h_{e}{|}^{2})\int_{|h_{e}{|}^{2}}^{\infty }\ln\left(\frac{1+\frac{\rho |h_{m}{|}^{2}}{|h_{p}{|}^{2}}}{1+\frac{\rho |h_{e}{|}^{2}}{|h_{p}{|}^{2}}}\right)\\ \; & \times f_{|h_{m}{|}^{2}}\left(\right|h_{m}{|}^{2})d|h_{m}{|}^{2}d{\left|h_{e}\right|}^{2}. \end{array}$$

*Substituting $|h_{m}{|}^{2}=\lambda_{m}x$ and $|h_{e}{|}^{2}=\lambda_{e}y$ into (36), and applying the scenario when the average power gains of both the main and wiretap channels go to infinity (i.e., $\lambda_{m}\to \infty$ and $\lambda_{e}\to \infty$), and after some involved mathematical simplifications, we can express (36) as*

(37) $$\begin{array}{c} {\bar{C}}_{sec}^{I}\;\approx \;\frac{4}{\ln(2)}\;\int_{0}^{\infty }\;\;\;\;\int_{\frac{\lambda_{e}}{\lambda_{m}}y}^{\infty }\;\;\;\;\;\ln\left(\;\frac{\lambda_{m}x}{\lambda_{e}y}\right)K_{0}\left(2\sqrt{x}\right)K_{0}\left(2\sqrt{y}\right)dxdy. \end{array}$$

*Now, by using (eq. (6.561.8)) of [48] and the transformations $-\frac{z^{a}}{\pi (z+1)}\ln\left(z\right)=G_{3,3}^{2,2}\left({z|}_{a,a,a+0.5}^{a,a,a+0.5}\right)$ (eq. (07.34.03.0919.01)) of [49] and $K_{\nu }\left(\sqrt{z}\right)=\frac{1}{2}G_{0,2}^{2,0}\left(\frac{z}{4}|\frac{\nu }{2},\frac{\nu }{2}\right)$, (eq. (03.04.26.0009.01)) of [49], into (37), and then simplifying it via (eq. (07.34.21.0011.01)) of [49] and (eq. (07.34.21.0081.01)) of [49], and after some algebraic simplifications, the ESC expression can be obtained, as shown in in (37), when $\lambda_{m}\to \infty$ and $\lambda_{e}\to \infty$. We skipped the detailed analysis here for brevity. Moreover, it can be seen from (37) that the ESC improves with $\lambda_{m}$ and $\lambda_{e}$; however, an error floor can be seen in the ESC performance in the high $\lambda_{m}$ and $\lambda_{e}$ regime. This is because of the reason that the channel strengths of both main link and wiretap link are improved simultaneously. This behavior is also shown numerically in Section 5.*

**Remark** **14.** *We can further express (37) as*

(38) $$\begin{array}{cc} {\bar{C}}_{sec}^{I} & \approx \frac{4}{\ln(2)}[\int_{0}^{\infty }\;\;\int_{\frac{\lambda_{e}}{\lambda_{m}}y}^{\infty }\;\;\;\;\ln\left(\frac{x}{y}\right)K_{0}\left(2\sqrt{x}\right)K_{0}\left(2\sqrt{y}\right)dxdy\\ \; & +\ln\left(\frac{\lambda_{m}}{\lambda_{e}}\right)\;\int_{0}^{\infty }\;\;\int_{\frac{\lambda_{e}}{\lambda_{m}}y}^{\infty }\;\;\;\;K_{0}\left(2\sqrt{x}\right)K_{0}\left(2\sqrt{y}\right)dxdy]. \end{array}$$

*It can be seen from (38) that both the integrals are consistent and can easily be evaluated. Therefore, we can conclude that the asymptotic ESC follows the scaling law of $\Theta \left(\ln\left(\frac{\lambda_{m}}{\lambda_{e}}\right)\right)$ as $\frac{\lambda_{m}}{\lambda_{e}}$ increases and thus depends on the relative channel strengths of ST→SR and ST→E links, which is also demonstrated via numerical results in Section 5.*

### 4.2. Strategy II: Combined Power Constraint of the Interference at the PR and Maximum Transmit Power at the ST

The ESC under Strategy II can be formulated as

(39) $$\begin{array}{cc} \; & {\bar{C}}_{sec}^{II}=E\left[\log_{2}\left(1+\min\left(\frac{\rho }{|h_{p}{|}^{2}},\rho_{1}\right){\left|h_{m}\right|}^{2}\right)-\log_{2}\left(1+\min\left(\frac{\rho }{|h_{p}{|}^{2}},\rho_{1}\right){\left|h_{e}\right|}^{2}\right)\right]\\ \; & =\underset{≜\;{\bar{C}}_{sec,1}^{II}}{\underset{︸}{E\left[\log_{2}\left(1+\frac{\rho |h_{m}{|}^{2}}{|h_{p}{|}^{2}}\right)-\log_{2}\left(1+\frac{\rho |h_{e}{|}^{2}}{|h_{p}{|}^{2}}\right)|{\left|h_{p}\right|}^{2}>\frac{\rho }{\rho_{1}}\right]}}\\ \; & +\underset{≜\;{\bar{C}}_{sec,2}^{II}}{\underset{︸}{E\left[\log_{2}\left(1+\rho_{1}{\left|h_{m}\right|}^{2}\right)-\log_{2}\;\left(1+\rho_{1}{\left|h_{e}\right|}^{2}\right)|{\left|h_{p}\right|}^{2}\leq \frac{\rho }{\rho_{1}}\right]}}, \end{array}$$

which can be simplified as per the following theorem.

**Theorem** **5.** *The exact expression of ESC under Strategy II over double-Rayleigh fading channels using (39) is given by*

(40) $$\begin{array}{cc} \; & {\bar{C}}_{sec}^{II}={\bar{C}}_{sec,1}^{II}+{\bar{C}}_{sec,2}^{II}, \end{array}$$

*where*

$$\begin{array}{cc} \; & {\bar{C}}_{sec,1}^{II}=\frac{1}{\ln(2)}\sum_{i=1}^{U}\frac{w_{i}e^{t_{i}}}{\lambda_{p}}\left[\frac{t_{i}}{\rho \lambda_{m}}G_{2,4}^{4,1}\left(\frac{t_{i}}{\rho \lambda_{m}}{|}_{0,0,-1,-1}^{-1,0}\right)-t_{i}^{\frac{3}{2}}\;\frac{\sqrt{\lambda_{m}}+\sqrt{\lambda_{e}}}{\rho^{\frac{3}{2}}\lambda_{m}\lambda_{e}}S\left(\frac{t_{i}}{\rho }\right)\right]\\ \; & \times G_{0,2}^{2,0}\left(\frac{t_{i}}{\lambda_{p}}|0,0\right)-\frac{2}{\ln(2)}[\sum_{k=1}^{N}\frac{g_{k}\rho }{\rho_{1}^{2}\lambda_{m}\lambda_{p}}G_{0,2}^{2,0}\left(\frac{\rho r_{k}^{2}}{\rho_{1}\lambda_{p}}|0,0\right)G_{2,4}^{4,1}\left(\frac{r_{k}^{2}}{\rho_{1}\lambda_{m}}{|}_{0,0,-1,-1}^{-1,0}\right) \end{array}$$

(41) $$\begin{array}{cc} \; & -\frac{\sqrt{\lambda_{m}}+\sqrt{\lambda_{e}}}{\lambda_{m}\lambda_{e}}\frac{\rho }{\rho_{1}^{\frac{5}{2}}}\sum_{k_{1}=1}^{M}\frac{g_{k_{1}}}{\lambda_{p}}G_{0,2}^{2,0}\left(\frac{\rho r_{k_{1}}^{2}}{\rho_{1}\lambda_{p}}|0,0\right)S\left(\frac{r_{k_{1}}^{2}}{\rho_{1}}\right)],\\ \; & {\bar{C}}_{sec,2}^{II}=\frac{1}{\ln(2)}\left[\frac{1}{\rho_{1}\lambda_{m}}G_{2,4}^{4,1}\left(\frac{1}{\rho_{1}\lambda_{m}}{|}_{0,0,-1,-1}^{-1,0}\right)-\frac{\sqrt{\lambda_{m}}+\sqrt{\lambda_{e}}}{\rho_{1}^{\frac{3}{2}}\lambda_{m}\lambda_{e}}S\left(\frac{1}{\rho }\right)\right] \end{array}$$

(42) $$\begin{array}{cc} \; & \times \left[1-\frac{2\sqrt{\rho }}{\sqrt{\rho_{1}\lambda_{p}}}K_{1}\left(\frac{2\sqrt{\rho }}{\sqrt{\rho_{1}\lambda_{p}}}\right)\right], \end{array}$$

*where $w_{i}=\frac{t_{i}}{{\left(\left(U+1\right)L_{U+1}\left(t_{i}\right)\right)}^{2}}$ and $t_{i}$, (i=1,⋯,U) are the weights and zeros of U−order Gauss–Laguerre polynomial (i.e., $L_{U}\left(t\right)$) [54], $g_{k}={\left\{\sum_{j=0}^{N-1}{\left[q_{j}\left(r_{k}\right)\right]}^{2}\right\}}^{-1}$ and $r_{k}$, (k=1,⋯,N) are the weights and zeros of N−order Gauss–Lobatto’s polynomial [54], respectively, $q_{N}\left(r\right)=\sqrt{2N+4}P_{N}^{\left(3,0\right)}\left(1-2r\right)$ with $P_{N}^{\left(3,0\right)}$ as Jacobi polynomial, and $g_{k_{1}}={\left\{\sum_{j_{1}=0}^{M-1}{\left[q_{j_{1}}\left(r_{k_{1}}\right)\right]}^{2}\right\}}^{-1}$ and $r_{k_{1}}$, $\left(k_{1}=1,\cdots ,M\right)$ are the weights and zeros of M−order Gauss–Lobatto’s polynomial, respectively, $q_{M}\left(r\right)=\sqrt{2M+5}P_{M}^{\left(4,0\right)}\left(1-2r\right)$ with $P_{M}^{\left(4,0\right)}$ as Jacobi polynomial.*

**Proof.**  See Appendix D for the detailed proof. □

**Remark** **15.** *It should be noted that (40) involves powers, exponential, Meijer-G functions, modified Bessel function of second kind, and extended generalized bivariate Meijer-G functions, consisting of network parameters $I_{p}$, Q, η, $\Omega_{m}$, $\Omega_{e}$, and $\Omega_{p}$, which can be efficiently calculated via Mathematica software. We can achieve an arbitrary accurate approximation by appropriately selecting the values of U, N, and M.*

**Remark** **16.** *Using (39) for $|h_{m}{|}^{2}\geq {\left|h_{e}\right|}^{2}$, the ESC under strategy II can be expressed as*

(43) $$\begin{array}{cc} {\bar{C}}_{sec}^{II} & =\frac{1}{\ln(2)}\int_{\frac{\rho }{\rho_{1}}}^{\infty }\;[\int_{0}^{\infty }f_{|h_{e}{|}^{2}}\left(\right|h_{e}{|}^{2})\;\;\int_{|h_{e}{|}^{2}}^{\infty }\;\;\;\ln\left(\frac{1+\frac{\rho |h_{m}{|}^{2}}{|h_{p}{|}^{2}}}{1+\frac{\rho |h_{e}{|}^{2}}{|h_{p}{|}^{2}}}\;\right)\\ \; & \times f_{|h_{m}{|}^{2}}\left(\right|h_{m}{|}^{2})d|h_{m}{|}^{2}d|h_{e}{|}^{2}]f_{|h_{p}{|}^{2}}\left(\right|h_{p}{|}^{2})d|h_{p}{|}^{2}\\ \; & +\frac{1}{\ln(2)}\int_{\frac{\rho }{\rho_{1}}}^{\infty }[\int_{0}^{\infty }f_{|h_{e}{|}^{2}}\left(\right|h_{e}{|}^{2})\int_{|h_{e}{|}^{2}}^{\infty }\ln\left(\frac{1+\rho_{1}{\left|h_{m}\right|}^{2}}{1+\rho_{1}{\left|h_{e}\right|}^{2}}\right)\\ \; & \times f_{|h_{m}{|}^{2}}\left(\right|h_{m}{|}^{2})d|h_{m}{|}^{2}d|h_{e}{|}^{2}\;]\;f_{|h_{p}{|}^{2}}\left(\right|h_{p}{|}^{2})d|h_{p}{|}^{2}. \end{array}$$

Substituting $|h_{m}{|}^{2}=\lambda_{m}x$ and $|h_{e}{|}^{2}=\lambda_{e}y$ into (43), and under the scenario when $\lambda_{m}\to \infty$ and $\lambda_{e}\to \infty$, and after some mathematical simplifications, we can express (43) as

(44) $$\begin{array}{cc} {\bar{C}}_{sec}^{II} & \approx \frac{4}{\ln(2)}\int_{0}^{\infty }\int_{\frac{\lambda_{e}}{\lambda_{m}}y}^{\infty }\ln\left(\frac{\lambda_{m}x}{\lambda_{e}y}\right)K_{0}\left(2\sqrt{x}\right)K_{0}\left(2\sqrt{y}\right)dxdy\\ \; & \times \left[\int_{\frac{\rho }{\rho_{1}}}^{\infty }f_{|h_{p}{|}^{2}}\left(\right|h_{p}{|}^{2})d|h_{p}{|}^{2}+\int_{0}^{\frac{\rho }{\rho_{1}}}f_{|h_{p}{|}^{2}}\left(\right|h_{p}{|}^{2})d|h_{p}{|}^{2}\right]\\ \; & \approx \frac{4}{\ln(2)}\int_{0}^{\infty }\int_{\frac{\lambda_{e}}{\lambda_{m}}y}^{\infty }\ln\left(\frac{\lambda_{m}x}{\lambda_{e}y}\right)K_{0}\left(2\sqrt{x}\right)K_{0}\left(2\sqrt{y}\right)dxdy. \end{array}$$

Further, we can simplify (44) by using (eq. (6.561.8)) of [48], (eq. (07.34.03.0919.01)) of (eq. (03.04.26.0009.01)) of [49], (eq. (07.34.21.0011.01)) of [49], and (eq. (07.34.21.0081.01)) of [49], whose detailed analysis is skipped here for brevity. From (44), one can observe that the ESC performance increases with the increased in $\lambda_{m}$ and $\lambda_{e}$ but saturates in the high $\lambda_{m}$ and $\lambda_{e}$ regime because of the simultaneous improvement in the channel strengths of both the main link and the wiretap link, as shown numerically in Section 5.

**Remark** **17.** *We can further express (44) as*

(45) $$\begin{array}{cc} {\bar{C}}_{sec}^{II} & \approx \frac{4}{\ln(2)}[\int_{0}^{\infty }\int_{\frac{\lambda_{e}}{\lambda_{m}}y}^{\infty }\ln\left(\frac{x}{y}\right)K_{0}\left(2\sqrt{x}\right)K_{0}\left(2\sqrt{y}\right)dxdy\\ \; & +\ln\left(\frac{\lambda_{m}}{\lambda_{e}}\right)\int_{0}^{\infty }\int_{\frac{\lambda_{e}}{\lambda_{m}}y}^{\infty }K_{0}\left(2\sqrt{x}\right)K_{0}\left(2\sqrt{y}\right)dxdy]. \end{array}$$

The integrals in (45) are consistent and can readily be simplified. Moreover, we can see from (45) that the asymptotic ESC follows the scaling law of $\Theta \left(\ln\left(\frac{\lambda_{m}}{\lambda_{e}}\right)\right)$ as $\frac{\lambda_{m}}{\lambda_{e}}$ increases, as shown numerically in Section 5.

## 5. Numerical Results and Discussion

In this section, we provide the numerical and simulation results to validate the effectiveness of our derived analytical findings under the consideration of Strategy I and Strategy II. To demonstrate, we plot various curves by varying the channel strengths ($\Omega_{m}$, $\Omega_{e}$, and $\Omega_{p}$) of ST→SR, ST→E, and ST→PR links. Note that a path-loss channel modeling can also be adopted, where the average channel power gains of all channels can be denoted as $\Omega_{ı}=d_{ı}^{-\nu }$, for ı={m,e,p}, where ν denotes the path-loss exponent, and $d_{ı}$ is the euclidean distance between the two nodes having the coordinates $\left(x_{ı},y_{ı}\right)$ and $\left(x_{ȷ},y_{ȷ}\right)$, for ı={m,e,p}, ȷ={m,e,p}, and ı≠ȷ. Such modeling indicates that, $\Omega_{ı}\to \infty$ correspond to $d_{ı}\to 0$, which implies that the nodes are located close to each other, whereas $\Omega_{ı}\to 0$ correspond to $d_{ı}\to \infty$, which indicates that two nodes are located far away from each other. Furthermore, we consider the Gauss–Laguerre polynomial order U=30 and Gauss–Lobatto’s polynomial order N=M=50, to obtain precise results.

### 5.1. SOP Performance under Strategies I and II

**Figure 5:**
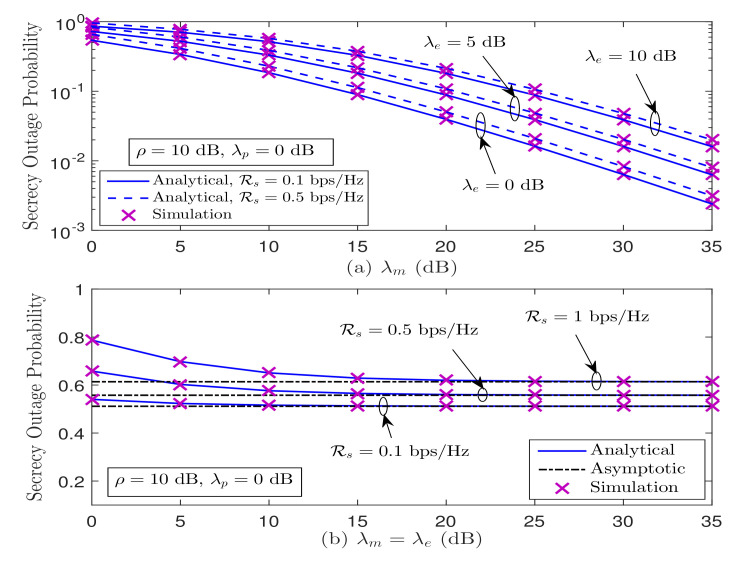
SOP performance for the considered system under Strategy I.

In Figure 5, we plot the SOP performance versus $\lambda_{m}$ and $\lambda_{e}$ for Strategy I. In Figure 5a, we show the SOP performance versus $\lambda_{m}$ for different values of $\lambda_{e}$ and $R_{s}$, when ρ=10 dB and $\lambda_{p}=0$ dB. We can observed from Figure 5a that the derived analytical results are in good agreement with the simulation results over the entire range of $\lambda_{m}$. Further, we can see that the SOP performance improves as $\lambda_{m}$ increases, and an effective secrecy diversity order of 1 can be verified irrespective of $\lambda_{e}$ and $R_{s}$, as also analytically demonstrated in Section 3-A. As expected, the SOP performance deteriorates with the improvement in wiretap channel strength $\lambda_{e}$, regardless of $\lambda_{m}$ and $R_{s}$. In Figure 5b, we demonstrate the SOP performance with average channel gains of both the main and wiretap links simultaneously varying (i.e., $\lambda_{m}=\lambda_{e}$ dB) for various values of $R_{s}$, when ρ=10 dB and $\lambda_{p}=0$ dB. From which, it is observed that the SOP decreases as average channel gains ($\lambda_{m}=\lambda_{e}$ dB) increase, but saturates in the medium-to-high average channel gains regime, regardless of $R_{s}$. This observation is also aligned with the derived asymptotic SOP results presented in (23), which depends on the fixed ratio $\frac{\lambda_{e}}{\lambda_{m}}$. In addition, we can see that the SOP performance decreases with the improvement in $R_{s}$, since more power is needed to achieve the higher value of $R_{s}$.

**Figure 6:**
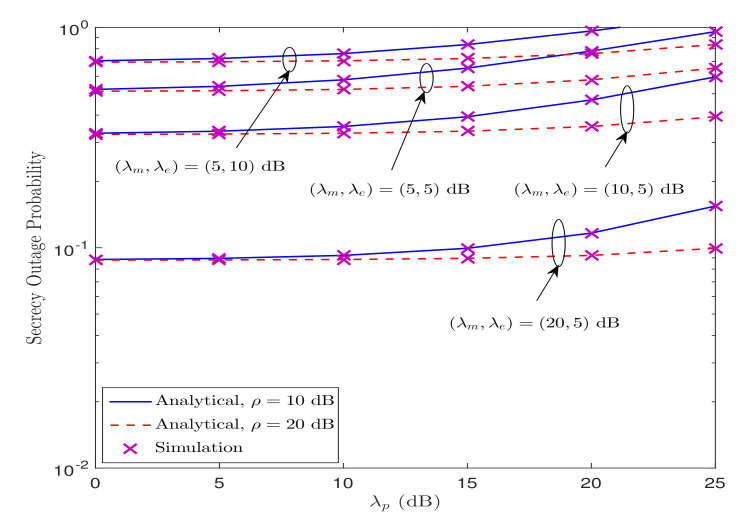
Impact of PR on the SOP performance under Strategy I.

Figure 6 illustrates the impact of PR on the SOP performance for various values of ρ, $\lambda_{m}$, and $\lambda_{e}$, when $R_{s}=0.1$ bps/Hz. We can see from this figure that the SOP performance deteriorates as $\lambda_{p}$ increases, irrespective of ρ, $\lambda_{m}$, and $\lambda_{e}$. This is because of the reason that the transmit power at ST decreases as $\lambda_{p}$ increases. Moreover, for fixed value of ρ, the SOP performance improves when the legitimate channel quality is better than the wiretap channel quality, i.e., $\lambda_{m}>\lambda_{e}$, and vice versa. In addition, the SOP performance improves as ρ increases, i.e., the performance is better for ρ=20 compared to ρ=10 dB. This is due to the fact that an increase in ρ allows ST to transmit at a higher power level without interfering with the PR.

**Figure 7:**
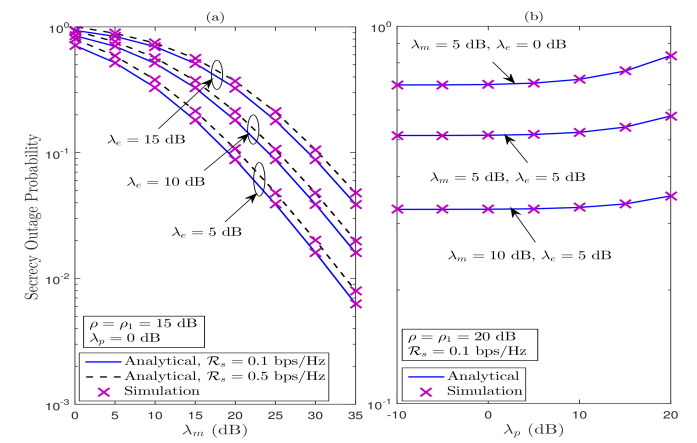
SOP performance for the considered system under Strategy II, (**a**) SOP versus $\lambda_{m}$, and (**b**) SOP versus $\lambda_{p}$.

In Figure 7, we demonstrate the impact of $\lambda_{m}$, $\lambda_{e}$, and $\lambda_{p}$ on the SOP performance under Strategy II. It can be observed from Figure 7 that the analytical results match perfectly with the simulation results, which corroborate the correctness of our derived theoretical findings. Figure 7a illustrates the SOP performance versus $\lambda_{m}$ for various values of $\lambda_{e}$ and $R_{s}$, when $\rho =\rho_{1}=15$ dB and $\lambda_{p}=0$ dB. We can observe that the SOP performance enhances as $\lambda_{m}$ increases; however, it decreases with the improvement in $\lambda_{e}$. Moreover, the effective secrecy diversity order of 1 can also be achieved for different set of involved parameters. As expected, the higher $R_{s}$ results into the SOP performance degradation. In Figure 7b, we show the impact of PR on the SOP performance under Strategy II for various values of $\lambda_{m}$ and $\lambda_{e}$, when $R_{s}=0.1$ bps/Hz and $\rho =\rho_{1}=20$ dB. It can be seen that the SOP performance degrades as $\lambda_{p}$ increases, since power at ST reduces as $\lambda_{p}$ improves. In addition, the SOP performance significantly improves if $\lambda_{m}>\lambda_{e}$, for all values of $\lambda_{p}$.

**Figure 8:**
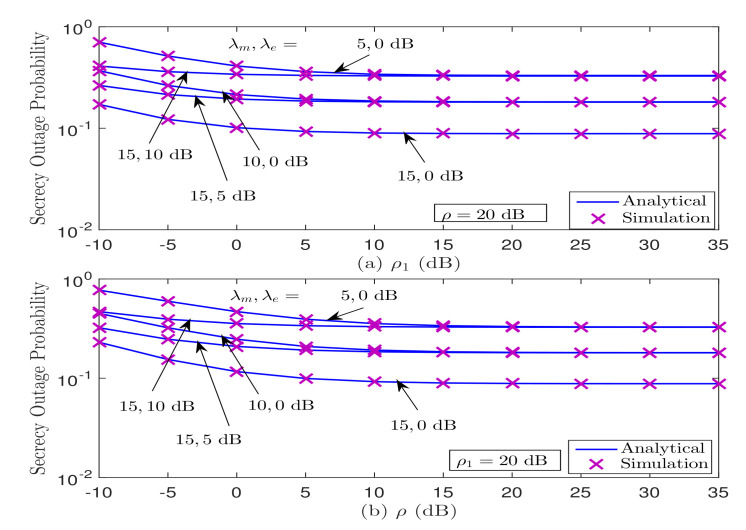
Impact of maximum tolerable interference level $\left(\rho =I_{P}/N_{0}\right)$ and maximum transmit power constraint $\left(\rho_{1}=Q/N_{0}\right)$ on the SOP performance under Strategy II.

In Figure 8, we illustrate the impact of maximum tolerable interference level $I_{P}$ and maximum transmit power constraint *Q* on the SOP performance behavior under Strategy II. It can be observed from Figure 8a,b that the SOP performance deteriorates when $\lambda_{e}>\lambda_{m}$ in the low ρ and $\rho_{1}$ regimes; however, it saturates as ρ and $\rho_{1}$ increase (i.e., in the medium-to-high ρ and $\rho_{1}$ regimes). The secrecy floor in Figure 8a occurs because the SOP is independent of maximum secondary transmitter power, $\rho_{1}≜\frac{Q}{N_{0}}$, in the high $\rho_{1}$, and only depends on fixed ρ, as also theoretically verified in (32). Furthermore, the secrecy floor is observed in Figure 8b due to the limited impact of ρ on the SOP in the high ρ regime, since the SOP depends on the fixed ratio $\frac{\rho }{\rho_{1}}$, as also analytically validated in (31). In other words, the secrecy floor occurs in the high ρ region since the SNR both the legitimated link and wiretap link is improved simultaneously. In addition, Figure 8a,b implies that the secrecy diversity order reduces to zero, which is perfectly aligned with the theoretical findings obtained in Section 3-B.

### 5.2. ESC Performance under Strategies I and II

**Figure 9:**
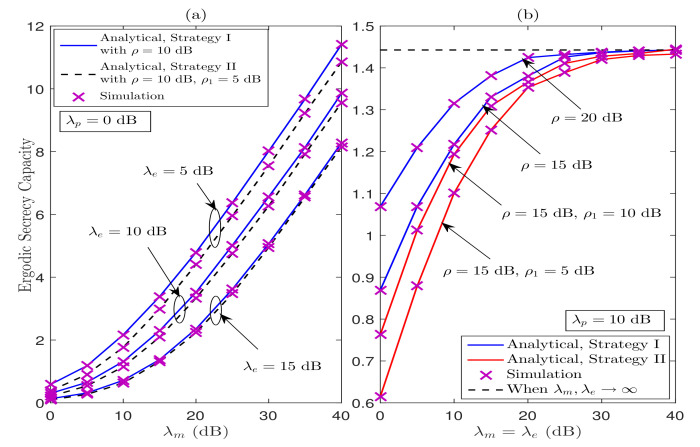
ESC performance for the considered system under Strategy I and Strategy II.

Figure 9 illustrates the ESC curves for various values of $\lambda_{m}$ and $\lambda_{e}$ under Strategy I and Strategy II. We can observe that the analytical ESC results under Strategies I and II are in good agreement with the simulation results over the entire regime of $\lambda_{m}$ and $\lambda_{e}$. We can observe from Figure 9a that the ESC performance increases as $\lambda_{m}$ increases under both the considered strategies. In addition, the ESC performance decreases significantly as the quality of wiretap link improves. Further, in Figure 9b, the ESC performance increases as $\lambda_{m}=\lambda_{e}$ dB increases; however, the performance saturates in the high $\lambda_{m}=\lambda_{e}$ dB regime, which is aligned with the theoretical findings obtained in Section 4. The reason behind this behavior is that the quality of wiretap channel increases in the same proportion as of legitimate channel, hence restricting further improvement in the ESC performance.

**Figure 10:**
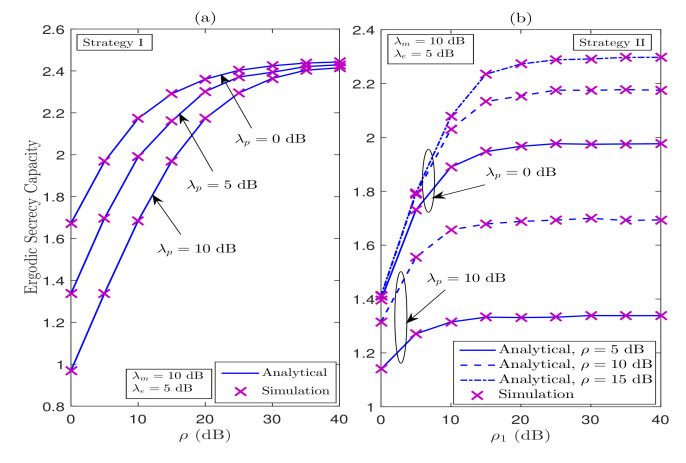
Impact of maximum tolerable interference level $\left(\rho =I_{P}/N_{0}\right)$ and maximum transmit power constraint $\left(\rho_{1}=Q/N_{0}\right)$ on the ESC performance.

In Figure 10, we demonstrate the impact of primary user $\left(\lambda_{p}\right)$, maximum tolerable interference level $I_{P}$, and maximum transmit power constraint *Q* on the ESC performance of the considered system. Figure 10a shows the ESC performance versus ρ under Strategy I for various values of $\lambda_{p}$, when $\lambda_{m}=10$ dB and $\lambda_{p}=5$ dB. The ESC performance increases as ρ increases; however, a secrecy floor is observed in the medium-to-high regime of ρ. This is because of the fact that an increase in ρ benefits both the legitimate destination and the eavesdropper. Moreover, the performance significantly deteriorates as $\lambda_{p}$ increases for all values of ρ. This degradation in ESC performance is because of the fact that the power at ST reduces as $\lambda_{p}$ increases. Moreover, in Figure 10b, we plot the curves for ESC versus $\rho_{1}$ under Strategy II for various values of ρ and $\lambda_{p}$. We can observe from this figure that the ESC performance improves with $\rho_{1}$ when $\rho \geq \rho_{1}$ and saturates when $\rho <\rho_{1}$. In other words, the ESC is affected by the interaction of ρ and $\rho_{1}$. When ρ is smaller than $\rho_{1}$, the SOP is mainly affected by ρ, whereas when $\rho_{1}$ is smaller than ρ, then $\rho_{1}$ becomes the dominant factor. In addition, the secrecy floor behavior is also due to the fact that both the eavesdropper and the legitimate destination simultaneously extract the same benefits of increased transmit powers. Further, Figure 10b under Strategy II reveals that the ESC performance is better for lower values of $\lambda_{p}$ than that of the one with higher values of $\lambda_{p}$.

**Figure 11:**
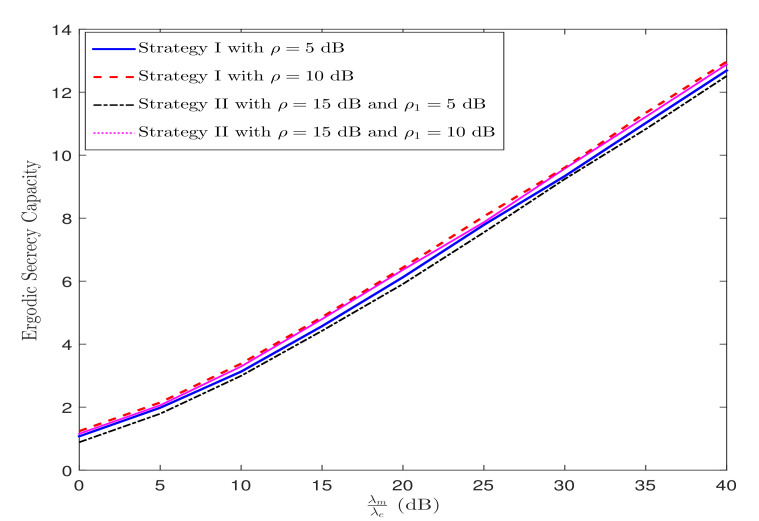
ESC performance versus $\frac{\lambda_{m}}{\lambda_{e}}$ under Strategy I and Strategy II.

Figure 11 illustrates the ESC versus $\frac{\lambda_{m}}{\lambda_{e}}$ for various ρ under Strategy I and for various ρ and $\rho_{1}$ under Strategy II, when $\lambda_{p}=0$ dB. It can be observed from Figure 11 that the ESC improves with increasing $\frac{\lambda_{m}}{\lambda_{e}}$. This is owing to a higher $\lambda_{m}$ than $\lambda_{e}$ implying a superior channel quality of the legitimate channel when compared to the channel quality of the eavesdropper. This behavior is also depicted theoretically in (38) for Strategy I and in (45) for Strategy II. I addition, it is seen that there is a linear relationship between the ESC growth rate and $\frac{\lambda_{m}}{\lambda_{e}}$ at high $\frac{\lambda_{m}}{\lambda_{e}}$.

## 6. Conclusions

This paper analyzed PHY-security in underlay CRVNs under spectrum-sharing constraints. Since all the nodes are in motion, the channels between the nodes are assumed to be modeled as double-Rayleigh fading. We assumed two different strategies to determine the transmit power of the secondary network. In Strategy I, the transmit power of the secondary transmitter is governed by the single-power constraint of the interference on the primary network, whereas in Strategy II, the transmit power of the secondary transmitter is governed by the combined power constraint of the interference on the primary network and the maximum transmission power at the secondary network. Under these two considered strategies, we deduced the exact SOP and ESC expressions for the considered system over double-Rayleigh fading channels. We also presented the asymptotic SOP analysis for the two considered strategies to reveal key insights into the system’s secrecy diversity order. It was demonstrated that the system can achieve a full secrecy diversity order of 1, when the average channel gain of main link goes to infinity with fixed average wiretap channel gain. Furthermore, from the ESC analysis, it is reveled that the ESC follows a scaling law of $\Theta \left(\ln\left(\frac{\Omega_{m}^{2}}{\Omega_{e}^{2}}\right)\right)$, when $\Omega_{m}$ and $\Omega_{e}$ go to infinity. We also verified our analytical findings via simulation studies. 

## Data Availability

This study did not report any data.

## Appendix A

### Proof of Theorem 1

By invoking the CDF of $|h_{e}{|}^{2}$ and the PDF of $|h_{m}{|}^{2}$ into (12), we can express the integral $I_{1}$ as

(A1) $$\begin{array}{c} I_{1}=I_{1a}-I_{1b}, \end{array}$$

(A2) $$\begin{array}{cc} \; & \mathrm{where}I_{1a}=\int_{\frac{(\eta -1)w}{\rho }}^{\infty }\frac{2}{\lambda_{m}}K_{0}\left(2\sqrt{\frac{y}{\lambda_{m}}}\right)dy, \end{array}$$

(A3) $$\begin{array}{cc} \; & I_{1b}=\frac{4}{\lambda_{m}\sqrt{\lambda_{e}}}\int_{\frac{(\eta -1)w}{\rho }}^{\infty }\sqrt{\frac{y}{\eta }-\frac{(\eta -1)w}{\eta \rho }}K_{0}\left(2\sqrt{\frac{y}{\lambda_{m}}}\right)K_{1}\left(\frac{2}{\sqrt{\lambda_{e}}}\sqrt{\frac{y}{\eta }-\frac{(\eta -1)w}{\eta \rho }}\right)dy. \end{array}$$

We can simplify $I_{1a}$ in (A2) by first applying the relation $\int_{\alpha }^{\infty }g\left(x\right)dx=\int_{0}^{\infty }g\left(x\right)dx-\int_{0}^{\alpha }g\left(x\right)dx$ and then making use of the facts (eq. (6.561.16)) of [48] and (eq. (6.561.8)) of [48], as presented in (14). Moreover, $I_{1b}$ in (A3) can be evaluated by first applying the change of variables $\frac{y}{\eta }-\frac{(\eta -1)w}{\eta \rho }=t$ and $\frac{\eta t}{\lambda_{m}}=r$ and the transformation $K_{\nu }\left(\sqrt{z}\right)=\frac{1}{2}G_{0,2}^{2,0}\left(\frac{z}{4}|\frac{\nu }{2},-\frac{\nu }{2}\right)$ (eq. (03.04.26.0009.01)) of [49] and then simplifying via (eq. (07.34.21.0082.01)) of [49], as shown in (15). Consequently, invoking (A2) and (A3) into (A1), we can obtain the inner integral $I_{1}$ as presented in (13).

## Appendix B

### Proof of Theorem 3

We can simplify $\Theta_{1}\left(\eta \right)$ in (24) as

(A4) $$\begin{array}{cc} \Theta_{1} & =\left[1-\int_{\frac{\eta -1}{\rho_{1}}}^{\infty }\;\;F_{|h_{e}{|}^{2}}\left(\frac{y}{\eta }-\frac{\eta -1}{\eta \rho_{1}}\right)f_{|h_{m}{|}^{2}}\left(y\right)dy\right]F_{|h_{p}{|}^{2}}\left(\frac{\rho }{\rho_{1}}\right)\\ \; & =[1-\int_{\frac{\eta -1}{\rho_{1}}}^{\infty }\frac{2}{\lambda_{m}}K_{0}\left(2\sqrt{\frac{y}{\lambda_{m}}}\right)dy\\ \; & +\frac{4}{\lambda_{m}\sqrt{\lambda_{e}}}\int_{\frac{\eta -1}{\rho_{1}}}^{\infty }\sqrt{\frac{y}{\eta }-\frac{\eta -1}{\eta \rho_{1}}}K_{1}\left(\frac{2}{\sqrt{\lambda_{e}}}\sqrt{\frac{y}{\eta }-\frac{\eta -1}{\eta \rho_{1}}}\right)\\ \; & \times K_{0}\left(2\sqrt{\frac{y}{\lambda_{m}}}\right)dy]\left[1-\frac{2\sqrt{\rho }}{\sqrt{\rho_{1}\lambda_{p}}}K_{1}\left(\frac{2\sqrt{\rho }}{\sqrt{\rho_{1}\lambda_{p}}}\right)\right]. \end{array}$$

The first integral in (A4) can easily be simplified with the facts that $\int_{\alpha }^{\infty }g\left(x\right)dx=\int_{0}^{\infty }g\left(x\right)dx-\int_{0}^{\alpha }g\left(x\right)dx$ (eq. (6.561.16)) of [48] and (eq. (6.561.8)) of [48], whereas we can simplify the second integral in (A4) by first applying the transformations of variables $\frac{y}{\eta }-\frac{\eta -1}{\eta \rho_{1}}=t$ and $\frac{\eta t}{\lambda_{m}}=r$ and then using (eq. (03.04.26.0009.01)) of [49] and (eq. (07.34.21.0082.01)) of [49]. Consequently, $\Theta_{1}\left(\eta \right)$ can be given in (26).

Moreover, we can express $\Theta_{2}\left(\eta \right)$ in (24) as

(A5) $$\begin{array}{cc} \;\Theta_{2}\left(\eta \right) & =\;\int_{\frac{\rho }{\rho_{1}}}^{\infty }\;\;\;\;f_{|h_{p}{|}^{2}}\left(y\right)dy-\;\;\int_{\frac{\rho }{\rho_{1}}}^{\infty }\;\;\{\;\int_{\frac{y(\eta -1)}{\rho }}^{\infty }\;\;F_{|h_{e}{|}^{2}}\left(\frac{x}{\eta }-\frac{y(\eta -1)}{\eta \rho }\right)\\ \; & \times f_{|h_{m}{|}^{2}}\left(x\right)dx\}f_{|h_{p}{|}^{2}}\left(y\right)dy, \end{array}$$

which can be further expressed after some simplifications as

(A6) $$\begin{array}{cc} \Theta_{2}\left(\eta \right) & =\frac{2\sqrt{\rho }}{\sqrt{\rho_{1}\lambda_{p}}}K_{1}\left(\frac{2\sqrt{\rho }}{\sqrt{\rho_{1}\lambda_{p}}}\right)-\int_{\frac{\rho }{\rho_{1}}}^{\infty }\{\int_{\frac{y(\eta -1)}{\rho }}^{\infty }f_{|h_{m}{|}^{2}}\left(x\right)\\ \; & \times F_{|h_{e}{|}^{2}}\left(\frac{x}{\eta }-\frac{y(\eta -1)}{\eta \rho }\right)dx\}f_{|h_{p}{|}^{2}}\left(y\right)dy. \end{array}$$

The inner integral in (A6) can be evaluated by invoking the CDF of $|h_{e}{|}^{2}$ and the PDF of $|h_{m}{|}^{2}$ and following the same steps as used to simplify the integrals in (A4). Then, invoking the result along with the PDF of $|h_{p}{|}^{2}$ into (A6), and after some simplifications, $\Theta_{2}\left(\eta \right)$ can be expressed as

(A7) $$\begin{array}{cc} \; & \Theta_{2}\left(\eta \right)=\frac{2\sqrt{\rho }}{\sqrt{\rho_{1}\lambda_{p}}}K_{1}\left(\frac{2\sqrt{\rho }}{\sqrt{\rho_{1}\lambda_{p}}}\right)\frac{4}{\lambda_{p}}\sqrt{\frac{\eta -1}{\rho \lambda_{m}}}\;\int_{\frac{\rho }{\rho_{1}}}^{\infty }\sqrt{y}K_{0}\left(\frac{2\sqrt{y}}{\sqrt{\lambda_{p}}}\right)K_{1}\left(2\sqrt{\frac{(\eta -1)y}{\rho \lambda_{m}}}\right)dy\\ \; & +\frac{2\sqrt{\lambda_{m}}}{\lambda_{p}\sqrt{\eta \lambda_{e}}}\sum_{k=0}^{\infty }\frac{{\left(-1\right)}^{k}}{k!}{\left(\frac{\eta -1}{\rho \lambda_{m}}\right)}^{k}G_{3,3}^{2,3}\left(\frac{\lambda_{m}}{\eta \lambda_{e}}{|}_{\frac{1}{2},-\frac{1}{2},k-\frac{1}{2}}^{-\frac{1}{2},k-\frac{1}{2},k-\frac{1}{2}}\right)\;\;\int_{\frac{\rho }{\rho_{1}}}^{\infty }y^{k}K_{0}\left(\frac{2\sqrt{y}}{\sqrt{\lambda_{p}}}\right)dy. \end{array}$$

The first integral (say $\chi_{1}$) in (A7) can be expressed by using the facts that $\int_{\alpha }^{\infty }g\left(x\right)dx=\int_{0}^{\infty }g\left(x\right)dx-\int_{0}^{\alpha }g\left(x\right)dx$ and (eq. (03.04.26.0009.01)) of [49] as

(A8) $$\begin{array}{cc} \; & \chi_{1}=\frac{1}{4}\int_{0}^{\infty }\;\;\;\;\sqrt{y}\;G_{0,2}^{2,0}\left(\frac{(\eta -1)y}{\rho \lambda_{m}}|\frac{1}{2},-\frac{1}{2}\right)G_{0,2}^{2,0}\left(\frac{y}{\lambda_{p}}|0,0\right)dy\\ \; & \;\;-\frac{1}{4}\int_{0}^{\frac{\rho }{\rho_{1}}}\;\;\;\;\sqrt{y}\;G_{0,2}^{2,0}\left(\;\frac{(\eta -1)y}{\rho \lambda_{m}}|\frac{1}{2},-\frac{1}{2}\right)G_{0,2}^{2,0}\left(\;\frac{y}{\lambda_{p}}|0,0\right)dy, \end{array}$$

where the first integral in (A8) can readily be simplified using (eq. (07.34.21.0011.01)) of [49], and the second integral in (A8) can be evaluated by first applying the transformation of variables $\frac{\rho_{1}y}{\rho }=t^{2}$ and then applying Guass–Lobatto’s quadrature integration [54]. Consequently, $\chi_{1}$ can be given by

(A9) $$\begin{array}{cc} \chi_{1} & =\frac{\lambda_{p}^{\frac{3}{2}}}{4}\;G_{2,2}^{2,2}\left(\frac{\left(\eta -1\right)\lambda_{p}}{\rho \lambda_{m}}{|}_{\frac{1}{2},-\frac{1}{2}}^{-\frac{1}{2},-\frac{1}{2}}\right)-\frac{\rho^{\frac{3}{2}}}{2\rho_{1}^{\frac{3}{2}}}\sum_{i=1}^{N}g_{i}t_{i}^{2}\\ \; & \times G_{0,2}^{2,0}\left(\frac{\left(\eta -1\right)t_{i}^{2}}{\rho_{1}\lambda_{m}}|\frac{1}{2},-\frac{1}{2}\right)G_{0,2}^{2,0}\left(\frac{\rho t_{i}^{2}}{\rho_{1}\lambda_{p}}|0,0\right). \end{array}$$

Furthermore, we can simplify the second integral (say $\chi_{2}$) of (A7) with the help of (eq. (03.04.26.0009.01)) of [49] and (eq. (07.34.21.0085.01)) of [49] as

(A10) $$\begin{array}{c} \chi_{2}={\left(\frac{\rho }{\rho_{1}}\right)}^{k+1}G_{1,3}^{3,0}\left(\frac{\rho }{\rho_{1}\lambda_{p}}{|}_{-k-1,0,0}^{-k}\right). \end{array}$$

Now, invoking (A9) and (A10) into (A7), and after some simplifications, we can express $\Theta_{2}\left(\eta \right)$ as presented in (27).

## Appendix C

### Proof of Theorem 4

With the aid of [56], we can express the ESC in (34) as

(A11) $$\begin{array}{cc} \; & {\bar{C}}_{sec}^{I}=\frac{1}{\ln(2)}\int_{0}^{\infty }[\underset{≜\;T_{1}}{\underset{︸}{\int_{0}^{\infty }\frac{y}{\rho }\ln\left(1+x\right)f_{|h_{m}{|}^{2}}\left(\frac{xy}{\rho }\right)F_{|h_{e}{|}^{2}}\left(\frac{xy}{\rho }\right)dx}}\\ \; & +\underset{≜\;T_{2}}{\underset{︸}{\int_{0}^{\infty }\frac{y}{\rho }\ln\left(1+x\right)f_{|h_{e}{|}^{2}}\left(\frac{xy}{\rho }\right)F_{|h_{m}{|}^{2}}\left(\frac{xy}{\rho }\right)dx}}\\ \; & -\underset{≜\;T_{3}}{\underset{︸}{\int_{0}^{\infty }\frac{y}{\rho }\ln\left(1+x\right)f_{|h_{e}{|}^{2}}\left(\frac{xy}{\rho }\right)dx}}]f_{|h_{p}{|}^{2}}\left(y\right)dy. \end{array}$$

By invoking the PDF of $|h_{m}{|}^{2}$ and the CDF of $|h_{e}{|}^{2}$ into $T_{1}$ of (A11), and applying the transformations $\ln\left(1+z\right)=G_{2,2}^{1,2}\left({z|}_{1,0}^{1,1}\right)$ (eq. (01.04.26.0003.01)) of [49] and $K_{\nu }\left(\sqrt{z}\right)=\frac{1}{2}G_{0,2}^{2,0}\left(\frac{z}{4}|\frac{\nu }{2},-\frac{\nu }{2}\right)$ (eq. (03.04.26.0009.01)) of [49], and then using (eq. (07.34.21.0011.01)) of [49] and (eq. (07.34.21.0081.01)) of [49], we can obtain $T_{1}$ as

(A12) $$\begin{array}{cc} T_{1} & =\frac{y}{\rho \lambda_{m}}G_{2,4}^{4,1}\left(\frac{y}{\rho \lambda_{m}}{|}_{0,0,-1,-1}^{-1,0}\right)-\frac{y^{\frac{3}{2}}}{\rho^{\frac{3}{2}}\lambda_{m}\sqrt{\lambda_{e}}}G_{2,2:0,2:0,2}^{2,1:2,0:2,0}\left({}_{-\frac{3}{2},-\frac{3}{2}}^{-\frac{3}{2},-\frac{1}{2}}|0,0|\frac{1}{2},-\frac{1}{2}|\frac{y}{\rho \lambda_{m}},\frac{y}{\rho \lambda_{e}}\right). \end{array}$$

On the same line, we can evaluate the integrals $T_{2}$ and $T_{3}$ in (A11), respectively, as

(A13) $$\begin{array}{cc} \; & T_{2}=\frac{y}{\rho \lambda_{e}}G_{2,4}^{4,1}\left(\frac{y}{\rho \lambda_{e}}{|}_{0,0,-1,-1}^{-1,0}\right)-\frac{y^{\frac{3}{2}}}{\rho^{\frac{3}{2}}\lambda_{e}\sqrt{\lambda_{m}}}G_{2,2:0,2:0,2}^{2,1:2,0:2,0}\left({}_{-\frac{3}{2},-\frac{3}{2}}^{-\frac{3}{2},-\frac{1}{2}}|0,0|\frac{1}{2},-\frac{1}{2}|\frac{y}{\rho \lambda_{m}},\frac{y}{\rho \lambda_{e}}\right), \end{array}$$

(A14) $$\begin{array}{cc} \; & T_{3}=\frac{y}{\rho \lambda_{e}}G_{2,4}^{4,1}\left(\frac{y}{\rho \lambda_{e}}{|}_{0,0,-1,-1}^{-1,0}\right). \end{array}$$

Now, invoking (A12), (A13), and (A14) alongwith the PDF of $|h_{p}{|}^{2}$ into (A11), and applying the transformation $K_{\nu }\left(\sqrt{z}\right)=\frac{1}{2}G_{0,2}^{2,0}\left(\frac{z}{4}|\frac{\nu }{2},-\frac{\nu }{2}\right)$ (eq. (03.04.26.0009.01)) of [49], it is observed that the solution of the resultant integral is tedious and intractable. To make the analysis tractable, we first multiply and divide the resultant integral by $e^{y}$, then simplifying it via Gauss–Laguerre numerical method [54]. Consequently, the ESC expression can be obtained, as shown in (35).

## Appendix D

### Proof of Theorem 5

The ${\bar{C}}_{sec,1}^{II}$ of (39) can be expressed in the integral form as

(A15) $$\begin{array}{cc} {\bar{C}}_{sec,1}^{II} & =\frac{1}{\ln(2)}\;\;\int_{\frac{\rho }{\rho_{1}}}^{\infty }[\underset{≜\;M_{1}}{\underset{︸}{\int_{0}^{\infty }\;\;\frac{y}{\rho }\ln\left(1+x\right)f_{|h_{m}{|}^{2}}\left(\frac{xy}{\rho }\right)F_{|h_{e}{|}^{2}}\left(\frac{xy}{\rho }\right)dx}}\\ \; & +\underset{≜\;M_{2}}{\underset{︸}{\int_{0}^{\infty }\frac{y}{\rho }\ln\left(1+x\right)f_{|h_{e}{|}^{2}}\left(\frac{xy}{\rho }\right)F_{|h_{m}{|}^{2}}\left(\frac{xy}{\rho }\right)dx}}\\ \; & -\underset{≜\;M_{3}}{\underset{︸}{\int_{0}^{\infty }\frac{y}{\rho }\ln\left(1+x\right)f_{|h_{e}{|}^{2}}\left(\frac{xy}{\rho }\right)dx}}]f_{|h_{p}{|}^{2}}\left(y\right)dy. \end{array}$$

The integrals $M_{1}$, $M_{2}$, and $M_{3}$ of (A15) can be evaluated by following the similar process as used to simplify $T_{1}$ in (A12), $T_{2}$ in (A13), and $T_{3}$ in (A14), respectively. Then, invoking the results along with the PDF of $|h_{p}{|}^{2}$ into (A15), and further using the facts that $\int_{\alpha }^{\infty }g\left(x\right)dx=\int_{0}^{\infty }g\left(x\right)dx-\int_{0}^{\alpha }g\left(x\right)dx$ and (eq. (03.04.26.0009.01)) of [49], and after some mathematical simplifications, we can express ${\bar{C}}_{sec,1}^{II}$ as

(A16) $$\begin{array}{cc} {\bar{C}}_{sec,1}^{II} & =\frac{1}{\ln(2)}\int_{0}^{\infty }\left[\frac{y}{\rho \lambda_{m}}G_{2,4}^{4,1}\left(\frac{y}{\rho \lambda_{m}}{|}_{0,0,-1,-1}^{-1,0}\right)-y^{\frac{3}{2}}\frac{\sqrt{\lambda_{m}}+\sqrt{\lambda_{e}}}{\rho^{\frac{3}{2}}\lambda_{m}\lambda_{e}}S\left(\frac{y}{\rho }\right)\right]\\ \; & \times \frac{1}{\lambda_{p}}G_{0,2}^{2,0}\left(\frac{y}{\lambda_{p}}|0,0\right)dy-\frac{1}{\ln(2)}\int_{0}^{\frac{\rho }{\rho_{1}}}\;\;\;\frac{1}{\lambda_{p}}G_{0,2}^{2,0}\left(\frac{y}{\lambda_{p}}|0,0\right)[\frac{y}{\rho \lambda_{m}}G_{2,4}^{4,1}\left(\frac{y}{\rho \lambda_{m}}{|}_{0,0,-1,-1}^{-1,0}\right)\\ \; & -y^{\frac{3}{2}}\frac{\sqrt{\lambda_{m}}+\sqrt{\lambda_{e}}}{\rho^{\frac{3}{2}}\lambda_{m}\lambda_{e}}S\left(\frac{y}{\rho }\right)]dy. \end{array}$$

The first integral in (A16) can be simplified by first multiplying and dividing it by $e^{y}$, and then applying the Guass–Laguarre quadrature method [54], whereas the second integral in (A16) can be evaluated by first using the transformation of variables $\frac{\rho_{1}y}{\rho }=r^{2}$ and then applying Guass–Lobatto’s integration method [54]. Consequently, the resultant expression of ${\bar{C}}_{sec,1}^{II}$ can be obtained, as presented in (41).

Furthermore, we can express ${\bar{C}}_{sec,2}^{II}$ of (39) as

(A17) $$\begin{array}{cc} {\bar{C}}_{sec,2}^{II} & =\frac{1}{\ln(2)}[\;\int_{0}^{\infty }\;\;\frac{1}{\rho_{1}}\ln\left(1+x\right)f_{|h_{m}{|}^{2}}\left(\frac{x}{\rho_{1}}\right)F_{|h_{e}{|}^{2}}\left(\frac{x}{\rho_{1}}\right)dx\\ \; & +\int_{0}^{\infty }\frac{1}{\rho_{1}}\ln\left(1+x\right)f_{|h_{e}{|}^{2}}\left(\frac{x}{\rho_{1}}\right)F_{|h_{m}{|}^{2}}\left(\frac{x}{\rho_{1}}\right)dx\\ \; & -\int_{0}^{\infty }\frac{1}{\rho_{1}}\ln\left(1+x\right)f_{|h_{e}{|}^{2}}\left(\frac{x}{\rho_{1}}\right)dx]F_{|h_{p}{|}^{2}}\left(\frac{\rho }{\rho_{1}}\right). \end{array}$$

By invoking the PDFs and CDFs of $|h_{m}{|}^{2}$ and $|h_{e}{|}^{2}$ and applying the transformations $\ln\left(1+z\right)=G_{2,2}^{1,2}\left({z|}_{1,0}^{1,1}\right)$ (eq. (01.04.26.0003.01)) of [49] and $K_{\nu }\left(\sqrt{z}\right)=\frac{1}{2}G_{0,2}^{2,0}\left(\frac{z}{4}|\frac{\nu }{2},-\frac{\nu }{2}\right)$ (eq. (03.04.26.0009.01)) of [49], and then simplifying the integrals with the help of (eq. (07.34.21.0011.01)) of [49] and (eq. (07.34.21.0081.01)) of [49], we can obtain ${\bar{C}}_{sec,2}^{II}$, as given in (42).
